# Practical pathway for the management of depression in the workplace: a Canadian perspective

**DOI:** 10.3389/fpsyt.2023.1207653

**Published:** 2023-09-05

**Authors:** Pratap Chokka, Ash Bender, Stefan Brennan, Ghalib Ahmed, Marc Corbière, David J. A. Dozois, Jeff Habert, John Harrison, Martin A. Katzman, Roger S. McIntyre, Yang S. Liu, Karen Nieuwenhuijsen, Carolyn S. Dewa

**Affiliations:** ^1^Department of Psychiatry, University of Alberta, Grey Nuns Hospital, Edmonton, AB, Canada; ^2^Work, Stress and Health Program, The Centre for Addiction and Mental Health, Department of Psychiatry, University of Toronto, Toronto, ON, Canada; ^3^Department of Psychiatry, University of Saskatchewan, Royal University Hospital, Saskatoon, SK, Canada; ^4^Department of Family Medicine and Psychiatry, University of Alberta, Edmonton, AB, Canada; ^5^Department of Education, Career Counselling, Université du Québec à Montréal, Centre de Recherche de l’Institut Universitaire en Santé Mentale de Montréal, Montréal, QC, Canada; ^6^Department of Psychology, University of Western Ontario, London, ON, Canada; ^7^Department of Family and Community Medicine, University of Toronto, Toronto, ON, Canada; ^8^Metis Cognition Ltd., Kilmington, United Kingdom; Centre for Affective Disorders, Institute of Psychiatry, Psychology and Neuroscience, King’s College, London, United Kingdom; Alzheimercentrum, AUmc, Amsterdam, Netherlands; ^9^START Clinic for the Mood and Anxiety Disorders, Toronto, ON, Canada; Department of Psychiatry, Northern Ontario School of Medicine, and Department of Psychology, Lakehead University, Thunder Bay, ON, Canada; ^10^Department of Psychiatry, University of Toronto, Toronto, ON, Canada; ^11^Department of Psychiatry, University of Alberta, Edmonton, AB, Canada; ^12^Department of Public and Occupational Health, Coronel Institute of Occupational Health, Amsterdam Public Health Research Institute, Amsterdam UMC, University of Amsterdam, Amsterdam, Netherlands; ^13^Department of Psychiatry and Behavioural Sciences, University of California, Davis, Davis, CA, United States

**Keywords:** mental health, major depressive disorder, occupational health, absenteeism, presenteeism, disability, return to work, workplace

## Abstract

Major depressive disorder (MDD) and other mental health issues pose a substantial burden on the workforce. Approximately half a million Canadians will not be at work in any week because of a mental health disorder, and more than twice that number will work at a reduced level of productivity (presenteeism). Although it is important to determine whether work plays a role in a mental health condition, at initial presentation, patients should be diagnosed and treated per appropriate clinical guidelines. However, it is also important for patient care to determine the various causes or triggers including work-related factors. Clearly identifying the stressors associated with the mental health disorder can help clinicians to assess functional limitations, develop an appropriate care plan, and interact more effectively with worker’s compensation and disability programs, as well as employers. There is currently no widely accepted tool to definitively identify MDD as work-related, but the presence of certain patient and work characteristics may help. This paper seeks to review the evidence specific to depression in the workplace, and provide practical tips to help clinicians to identify and treat work-related MDD, as well as navigate disability issues.

## 1. Burden and unmet needs around mental health in the workplace

### 1.1. Introduction and need for guidance in work-related MDD

Although the deleterious impact of major depressive disorder (MDD) on the workforce has been described over the past 15 years, there continues to be a critically high burden in terms of prevalence, lost work days, reduced productivity at work, higher unemployment risks, higher healthcare costs, and poorer quality of life (1–8). For example, studies have found that a 1% increase in unemployment is associated with a 1% increase in the suicide rate (9, 10). Thus, these data continue to support the need for action. The COVID-19 global pandemic, which affected both employment and mental health, further highlighted their interconnectedness (10–13). A systematic review including 18 studies found that, although there were conflicting findings, the majority of studies reported an increasing trend of suicidal attempts during compared to before the COVID-19 pandemic (14).

The aim of this report is to provide practical guidance to clinicians to assist them in assessing and treating work-related depression. The term “work-related” encompasses patients in whom work-related stressors have contributed to the development of their disorder, those who have developed their disorder subsequent to a physical or psychological work-related injury, and those who suffer an exacerbation of a pre-existing condition as a result of work-related stressors. Our target audience is clinicians, particularly primary care physicians in Canada, who are often the first line providers of treatment for MDD and for mitigating its impact on patients, their families, and their workplaces (15). There is a critical need for practical strategies to help clinicians efficiently and effectively provide the best possible care for their patients, as well as to help them to navigate the complex world of disability assessments, employee-employer relations, Workers’ Compensation Boards, and other reimbursement/insurance regulations. In addition, the information presented here may be of interest to employers.

Work-related MDD is increasingly requiring clinicians to serve as an intermediary between patients, employers, insurance providers and worker compensation systems. This has contributed to an urgent need for clinician-oriented guidance and education that will support accurate assessment and diagnosis of work-related MDD, and provide return to work principles, proper documentation procedures, effective workplace accommodations, and evidence-based treatments to specifically improve productivity.

Guidelines such as the national standards from the Mental Health Commission of Canada (MHCC) (16), and those from the World Health Organization (WHO) (17), are available to assist employers in creating and sustaining psychologically healthy work environments to prevent mental health issues in the workplace. However, there is little guidance to assist clinicians in assessing and treating work-related MDD. Canadian healthcare providers (HCPs) often lack specific training to assess for potential functional impairment due to mental health issues in a work context (18). In addition, the frustration that clinicians experience when treating workers struggling with a disability, and interacting with insurance providers and employers, can erode the therapeutic relationship and inhibit timely assessment and treatment (19).

### 1.2. Burden and impact of mental health disorders in the workplace

For Canadians with major depressive disorder (MDD), the prevalence of unemployment and disability has been reported to be as high as 30% (2, 20), and in 2016 mental health claims represented 70% of the total cost of all disability claims (2). In addition, the rate of presenteeism was about 60% among those with MDD and/or an anxiety disorder (21). The annual estimated costs attributable to absenteeism and presenteeism associated with MDD in Canada were $2.5 billion and $6.8 billion, respectively (22).

Whereas MDD and other mental disorders can lead to temporary or permanent loss of employment, unemployment can lead to further deterioration in mental health and reduced chances of gaining employment (23, 24). Long term work absence and disability are associated with a range of poor health outcomes including increased mortality rates (25, 26), especially from cardiovascular disease and suicide (9, 10, 25, 26), increased rates of pain, obesity, chronic illnesses, disability, and healthcare utilization and cost, and decreased quality of life have also been reported (25–27).

Recently, the COVID-19 global pandemic has had a substantial effect on both employment and mental health (10–12, 28). The associated increases in the incidence of psychosocial problems, unemployment and financial uncertainty substantially increased the risk of suicide (9, 10, 28). Therefore, addressing work-related mental health and preventing suicide is becoming a public and employer health priority.

### 1.3. Benefits of improving mental health management and workplace environments

A World Health Organization (WHO) study found that every dollar spent on treatment for depression and anxiety can result in as much as US$4.00 in better health and improved economic productivity (29). Better treatment could lead to faster recovery, which globally, could translate to an extra 43 million years of healthy life over 15 years (29). The Conference Board of Canada estimated that improving MDD treatment for employed Canadians would add approximately $32.3 billion to Canada’s economy (21).

Strategies in the workplace to improve psychological health and safety can have positive benefits for employers in areas such as risk mitigation, business outcomes or profitability, recruitment and retention, and organizational excellence and sustainability (16). Organizations with a proactive approach to maintaining their employees’ psychological wellbeing can benefit from reductions in absenteeism and presenteeism, as well as lower rates of turnover, disability, injury, conflict, grievance, and poor morale (16, 30). In a survey of 784 Canadians with a mental disorder, 83% required some form of workplace accommodation, yet only 30% had received it (31). This is unfortunate since it has been demonstrated that workplace accommodation can significantly reduce the risk of a persistent mental disorder (32). Improved productivity resulting from the appropriate accommodation and effective management of mental health issues can provide substantial cost-savings for employers (33).

### 1.4. Stigma of mental health disorders in the workplace

Although general awareness of mental disorders has improved, stigma remains a barrier to disclosing illness and obtaining care. A 2014 survey found that one-third of workers would not tell their employer if they had a mental health problem (34). One of their major concerns was fear of career damage, such as lost opportunities for promotion. In one survey, 26% of patients with MDD reported experiencing workplace discrimination, more than 50% expected to face discrimination, and 73% concealed their mental diagnosis (35). Similarly, a survey of the general public found that many do not regard depression as a valid reason for sickness absence (36). Stigma may also play a part in the workplace response to mental health injuries. When an employee suffers a physical work-related injury, they are usually given immediate treatment to manage their condition, but an immediate response to work-related mental health injuries can be impeded by the stigma around disclosing work-related mental health issues. Unfortunately, many work sites do not have a policy for safe disclosure (34).

## 2. Methods

The goal of this paper is to assist Canadian HCPs in the assessment and management of work-related depression. The guidance, developed by a committee of the Workplace Mental Health Network (WHMN), a non-profit organization focusing on the assessment and management of mental health disorders for clinicians, considered current evidence and the context of clinical challenges. Although this document focuses on a Canadian perspective, the general principles discussed have global implications and utility. This document is not a systematic review or a clinical guideline due to the paucity of evidence-based research in the literature to support some of the recommendations. However, the committee sought to provide guidance for clinicians, bridging gaps in the literature that lack strong evidence and addressing a real clinical problem, by using expert consensus.

Committee members were invited to participate in this process based on their area of interest, and their clinical and research expertise. Committee members were responsible for overseeing the development of the guidance and were assigned to review and provide critical feedback on each of the main topics based on their clinical expertise and research in mood disorders and occupational mental health. The topic assignments were as follows: introduction and summary sections (PC), guidance development and consensus methods (CD, PC, KN), assessment and diagnosis of work-related depression (AB, JH, GH, SB), pharmacotherapy (MK, RM, SB, JH, PC), psychotherapy (JH, MC, DD), and patient communication and advocacy (CD, AB, PC). In addition, each member of the committee reviewed the entire manuscript and provided feedback. Due to the impact of the COVID-19 pandemic, this guidance was developed through an iterative process involving videoconferencing, telephone, and email communication.

The committee initially reviewed and discussed the document, “Clinical guidelines for the diagnosis and management of work-related mental health conditions in general practice” developed in Australia, and published in 2019 (37). The quality of its guideline development methods was evaluated by two reviewers (CS, KN) using the AGREE II rating system (38). The Guideline Technical Report and the Administrative Report provided useful background information to support the quality of the guidelines (39, 40). The guideline development methods were rated according to the 23 AGREE II items and six dimensions. Each of the two reviewers independently scored each item (on a scale of 1 for the lowest quality to 7 for the highest quality), and disagreements were discussed until consensus on item scores was reached. Each dimension was then scored. The maximum dimension quality score was 100%, which indicated the percentage of items within the dimension that was rated at the highest level (score = 7). The AGREE II scores for six dimensions were (1): scope and purpose (100%) (2), stakeholder involvement (94%) (3), rigor of development (76%) (4), clarity of presentation (94%) (5), applicability (75%), and (6) editorial independence (100%). Of the six dimensions, the “rigor of development” dimension had one of the lowest scores. This was in large part related to little information about the point estimates and uncertainty of the estimates of the research upon which the recommendations were based and the mechanism to be used for updating the guidelines with new and emerging literature. This, however, did not have a meaningful impact on this committee’s work.

The Australian guidelines systematically reviewed the evidence published prior to mid-2017 and explicitly described the link between the recommendations and the supporting evidence. Although the guidelines reviewed most aspects of workplace depression treatments, it was mostly restricted to multi-pronged interventions focusing on specific workplace outcomes and did not thoroughly capture psychotherapy and pharmacotherapy interventions (37, 41). To address this gap, we conducted detailed literature searches for psychotherapy and pharmacotherapy interventions in work-related depression. The search terms included various versions of work or workplace, psychotherapy, and pharmacotherapy (including specific agents), with a supplemental search focusing on cognition and functioning (see Supplementary material S1). In addition, evidence was also identified and evaluated through the experts on the committee, and hand-searching the bibliographies of identified references.

In summary, the recommendations in this paper were based on the Australian guidelines and the author’s updated supplemental and targeted literature search to February 2023 using PubMed^1^ and expert consensus. Additionally, the recommendations incorporate real-world perspectives of Canadian and international clinicians with expertise in psychiatry, psychology, occupational medicine, family medicine, and public and occupational health. Patient cases that illustrate clinical situations encountered by HCPs when patients present with symptoms suggestive of a work-related mental health disorder are included in Supplementary material S2. A summary of clinical tips for assessing and managing workplace-related MDD is shown in Supplementary material S3.

## 3. Assessment and diagnosis of major depressive disorder–work factors contributing to mental health disorders

### 3.1. Screening and diagnosis of work-related mental health disorders

The diagnosis of a mental disorder should be based on a thorough clinical assessment, using The Diagnostic and Statistical Manual of Mental Disorders, Fifth Edition (DSM-5) criteria (42). Additionally, validated tools and scoring systems can be very helpful in screening, diagnosing, and assessing the severity of depressive symptoms. Primary care physicians are very accurate in ruling out mental disorders, but less so in identifying the presence of disorders (43, 44).

The Australian guidelines completed a GRADE review of the evidence for tools to diagnose and assess the severity of workers presenting with mental health symptoms (37, 39). They assessed 46 distinct tools including both patient- and clinician-completed questionnaires. Based on evidence from studies conducted in a work context, the Australian guideline recommended the Patient Health Questionnaire-9 (PHQ-9) for depression, and the Depression Anxiety Stress Scale (DASS) for anxiety disorders (37). However, based on the evidence and recommendations of other guidelines, they also recommended the Generalized Anxiety Disorder 7-item (GAD-7) for anxiety disorders (37). These tools are short and patient-completed. It is important to remember that while tools can improve accuracy, the diagnosis should always be confirmed with a comprehensive clinical assessment.

The differential diagnosis of MDD within the context of work should also consider other mental health disorders such as, but not limited to bipolar disorder, attention-deficit/hyperactivity disorder (ADHD), substance use disorders, and adjustment disorder as defined in the DSM-5 (42). Medical conditions, and in the case of work-related symptoms, the role of stress and burnout should also be considered. Useful screening tools to identify comorbid conditions associated with work-related MDD include the Mood Disorder Questionnaire (MDQ) for bipolar spectrum disorders (45), the ADHD Self-Report Scale (ASRS) (46), and Alcohol Use Disorders Identification Test (AUDIT) (47). Recently, validated, digital and machine-learning-based screening instruments such as EarlyDetect, promise some modern approaches in screening for MDD and other comorbid disorders such as ADHD and bipolar disorder (48–50). However, more research and clinical experience are required before such programs can replace traditional screening instruments.

In addition to symptom assessment in workplace MDD, it is important to evaluate how symptoms cause clinically relevant distress and impairment in social, occupational, or other areas of functioning. Assessing cognitive impairment is essential since it is significantly associated with social, occupational, and global functioning (51–53). Perceived cognitive function has been shown to have a greater impact on workplace dysfunction than the severity of depression among working adults with MDD (54, 55).

Abnormalities in executive function, working memory, attention, and psychomotor processing speed, have been shown to impact work performance in patients with MDD (54, 56, 57). In addition, improvements in cognitive symptoms are highly significantly correlated with improvements in workplace productivity (58).

Given the importance of cognitive impairment on functioning at work, home, and school, tools to assess cognition should be a part of the assessment of patients with work-related mental disorders. Examples of some of the validated tools for the assessment of cognitive functioning are shown in Table 1 (59–64). One caution when using these tools is the need for suitable norms, i.e., considering age, education, sex, and occupation.

**Table 1:** Examples of tools to assist clinicians in assessing cognition.

Scale	Rater	Overview
Perceived Deficit Questionnaire-Depression-20 (PDQ-D-20) (59, 60) PDQ-D-5 (61)	Patient	20 items assessing subjective cognitive difficulties: attention/concentration, prospective memory, retrospective memory, and planning/organization in MDD 5-item, PDQ-D-5 is more practical for use in clinical practice*
THINC-integrated tool (THINC-it) (62)	Patient	Computerized cognitive battery assessing both objective and subjective cognitive deficits in MDD 10–15 min, digital administration of 5 subtests: DSST, CRT, TMT-B, PDQ-D-5, and one-back working memory tool
British Columbia Cognitive Complaints Inventory (BC-CCI) (63)	Patient	6 items assessing perceived cognitive problems: concentration, memory, and thinking skills
Montreal Cognitive Assessment (MoCA) (64)	Clinician	7 subtests assessing objective cognitive deficits: visuospatial/ executive, naming, attention, language, abstraction, delayed recall, and orientation in patients with mild impairment or MDD

*Validated in healthy volunteers. BD, bipolar disorder; CRT, Choice Reaction Time; DSST, Digit Symbol Substitution Test; MDD, major depressive disorder; TMT-B, Trail Making Test B.

It is important to differentiate between subjective (e.g., as measured by the PDQ-D) and objective cognitive deficits [e.g., as measured by Digit Symbol Substitution Test (DSST)] (61, 65–67). Subjective cognition has been related to depression severity, whereas age has been found to be a greater predictor of objective cognitive ability (66, 67). Self-perceived cognitive function can be affected by emotional state and the severity of depressive symptoms to a greater extent than objective cognitive measures. In addition, subjectively-rated cognitive symptoms have been shown to be a stronger predictor of functional impairment, than have objectively-measured cognitive impairments (68). However, in one study of patients with MDD, 48% had subjective, and 64% had objective cognitive impairment (65). Overall, 80% of patients with MDD met the criteria for one or the other, but only 31% met the criteria for both. Therefore, it is important to measure both to get an accurate measure of cognitive functioning. The THINC-it tool combines objective and subjective cognitive testing (62).

#### 3.1.1. Determining the role of work in a mental health disorder

It is important for clinicians to assess work and patient factors that may contribute to workplace mental health disorders, specifically MDD. For example, in a 2013 survey of work-related mental disorders, 31–33% of claims were due to work pressure, and 18–27% to harassment or bullying at work (69). Work pressures were related to work deadlines, interpersonal conflicts with employers or colleagues, disciplinary actions, performance reviews, and promotion disappointments (69). Meta-analyzes of prospective and case–control studies have identified several workplace factors associated with depressive symptoms and absenteeism (Table 2) (70, 71).

**Table 2:** Work-related factors affecting the psychosocial wellbeing of workers (70, 71).

Factor
Low reward
Effort-reward imbalance
Job strain (high psychological demands and low decision latitude)
High psychological demands
Workplace bullying
Low support
Unfavorable social climate
Job insecurity
Conflicts/lack of work justice

In addition to workplace factors, certain patient characteristics may also indicate a higher likelihood of a work-related mental disorder, particularly among those who have experienced a physical or psychological work-related injury (37). These may include specific depressive symptoms of insomnia, low mood, anhedonia, and suicidal thoughts; chronic physical health problems; poor social and personal support system; history of past or current depression, anxiety, substance use, or other mental disorder. Thus, the relationship of a mental diagnosis to work should be based on the clinical assessment and assessment of the factors described in Table 2, and the timing of pressures, events, or changes in the workplace relative to symptom onset (16, 37, 70, 71).

Although screening tools to determine that a mental disorder is related to workplace factors are absent, tools to assess functioning and work impairment can be very useful in clinical assessment. Examples of some of the tools that have been validated for the assessment of functioning are shown in Table 3 (72–78). In addition, the Occupational Depression Inventory (ODI), a validated 10-item instrument, has shown utility to quantify the severity of work-attributed depressive symptoms (77, 78).

**Table 3:** Examples of tools to assist clinicians in assessing functioning or work productivity.

Scale	Rater	Overview
Sheehan Disability Scale (SDS) (72)	Patient	3 domains assessing work/school, social life/leisure, and family life/home responsibilities
World Health Organization Disability Assessment Schedule 2.0 (WHODAS) (73)	Patient, clinician	A generic instrument for assessing health status and disability across different cultures and settings 6 domains of functioning: cognition, mobility, self-care, getting along, life activities, and participation
University of California San Diego Performance-based Skills Assessment (UPSA) (74)	Clinician	5 domains of functioning: comprehension/planning, finance, transportation, household, and communication
Work Limitations Questionnaire (WLQ) (75)	Patient	25 questions assessing the level of work interference over the previous 2 weeks due to chronic conditions and their treatment 4 dimensions of demand: time, physical, mental-interpersonal, and output An 8-item version is commonly used to assess lost productivity
Work Productivity and Activity Impairment (WPAI) (76)	Clinician	6 questions yielding 4 score types to express impairment level over the previous 7 days: Absenteeism, presenteeism, work productivity loss, activity impairment
Occupational Depression Inventory (ODI) (77, 78)	Clinician	10 items (9 symptoms and a subsidiary question) to assess the severity of work-attributed depressive symptoms and establish provisional diagnoses of work-related MDD

#### 3.1.2. Distinguishing between burnout and MDD

Burnout is defined in the International Classification of Diseases 11^th^ Revision (ICD-11) as a syndrome resulting from chronic workplace stress, and it is specific to the occupational context (79). The three dimensions of burnout are exhaustion, mental distancing from one’s job, and reduced professional efficacy (79). There are no DSM-5 diagnostic criteria for burnout (42). Burnout resembles depression, and although some evidence suggests it is a distinct condition (80), this remains controversial (81). A systematic review examining the relationships between burnout and depression or anxiety concluded that the conditions were associated but did not conclusively overlap; burnout appeared to be a different construct (80). However, person-centered longitudinal analysis of symptom clusters found burnout and depression both develop in tandem at similar levels, suggesting conceptual similarity in the work context (82). A meta-analysis of 14 studies suggests the core symptoms of burnout, exhaustion, largely overlap with depression, compared to the putative dimension of detachment and efficacy (83). Both burnout and MDD have each been shown to predict suicide risk; however, burnout was not consistently associated with other suicide risk factors when MDD was present (84). In addition, people who suffer from burnout may present with many symptoms that look like those seen in patients with MDD (Table 4) (79, 80, 85).

**Table 4:** Comparing burn-out and major depressive disorder (MDD).

Major depressive disorder (MDD) (42)	Burnout (79, 80, 85)
Included as a diagnosis in the DSM-5 Depressed mood Loss of interest or pleasure Changes in weight and/or appetite Sleep problems Psychomotor agitation or retardation Fatigue or loss of energy Feelings of worthlessness or inappropriate guilt Impaired concentration, or indecisiveness Suicidal ideation	Not a diagnosis in the DSM-5 Listed as a syndrome, not a medical condition in the ICD-11 Feelings of energy depletion or exhaustion (particularly emotional exhaustion) Increased mental distance from one’s job, or feelings of negativism or cynicism related to one’s job (detachment) Reduced professional efficacy (sense of lack of accomplishment) Some symptoms may resemble those seen in MDD

Burnout is commonly assessed using the Maslach Burnout Inventory (MBI) (86, 87). However, because the symptoms included in burnout scales are very similar to some but not all of the symptoms that characterize depression, it is important to determine if patients presenting with burnout have MDD according to the DSM-5 criteria (88).

#### 3.1.3. When to consider malingering or factitious disorder

There are key differences between presentations of malingering, somatoform, and factitious disorder (Table 5) (42, 89, 90). It is important to be aware of and identify individuals who may have alternative gain and do not want to return to work, and to consider these other presentations in the differential diagnosis.

**Table 5:** Differences between somatoform disorder, factitious disorder, and malingering (42, 89, 90).

Diagnosis	Mechanism of illness production	Motivation for illness production
Somatic symptom disorder	**Unconscious** Excessive attention and treatment seeking for medical concerns	**Unconscious** No evidence of false information or deception
Factitious disorder	**Conscious** Falsification of physical or psychological symptoms	**Unconscious** Deception even in the absence of external rewards
Malingering	**Conscious** Intentional reporting of symptoms for personal gain (e.g., money, time off work)	

Malingering is not a medical diagnosis, and it can be difficult to identify with certainty. It is generally defined as fabricating or exaggerating symptoms (mental or physical) for secondary gains, such as financial compensation, obtaining drugs, avoiding obligations or discipline at school or work, or getting attention (91). Certain factors can increase the suspicion of malingering such as presentation in a medico-legal context, a discrepancy between self-reported stress or disability and medical findings, poor cooperation during an assessment, poor compliance or poor response to prescribed treatments, an unexpected escalation of symptoms, vagueness around prior management strategies, use of multiple different healthcare providers, and a history of antisocial behavior (90, 92). Studies have shown that patients who are malingering report more symptoms than those with confirmed MDD (89, 93). They may endorse suggested symptoms indiscriminately, including those that are atypical or rare, believing this will be more convincing of severe illness (93). Conversely, genuine patients report only the symptoms that they are really experiencing.

It is very challenging to identify malingering in the absence of a patient’s confession or contradictory objective information. The reliability of patient self-reports should be well documented in the patient’s records, and requests be made for an independent medical opinion to clarify the presence of genuine illness and impairment. Malingering might be suspected after conducting a thorough medical history, identifying inconsistency in symptom presentation, and observed mental status, and perhaps employing diagnostic tools such as the Minnesota Multiphasic Personality Inventory (MMPI-3) or the Personality Assessment Inventory (PAI) (94, 95). In the absence of proof of a conscious motivation for obvious personal gain, factitious disorder or somatic symptom disorder should also be considered after a thorough evaluation (Table 5) (42, 89, 90).

### 3.2. Identifying comorbid conditions

#### 3.2.1. Comorbid psychiatric conditions

Comorbid medical conditions and other mental disorders confer more disability, require more complex treatment approaches, and are associated with longer time to recovery, and a lower likelihood of achieving functional recovery. As a result, comorbid conditions can lead to greater work disability and more absenteeism than MDD alone (20, 96). Depression and anxiety are frequently comorbid, with around 50% of patients with MDD also suffering an anxiety disorder in their lifetime (97, 98). Comorbid anxiety has been associated with increased rates of suicide, poorer response to treatment, increased severity, increased risk of chronicity and recurrence, and differences in neurobiology compared to depression alone (99, 100). Substance use disorders are also common comorbidities in patients with MDD (101). Patients with depressive symptoms should be routinely assessed for anxiety, substance use, and suicide risk. Useful tools were mentioned previously.

#### 3.2.2. Comorbid non-psychiatric medical conditions

An estimated 2.2 million Canadian adults have both physical and mental comorbidity [Canadian Community Health Survey (CCHS), 2014] (102). The presence of a comorbid mental condition in patients with chronic medical conditions were associated with lower health-related quality of life (HRQoL), increased annual healthcare costs, and an increased risk of suicidal ideation (27, 102). Common comorbid conditions included hypertension, cancer, post-stroke, arthritis, cataracts, chronic obstructive pulmonary disease, diabetes, epilepsy, heart disease, pain, migraine, and thyroid disease (27, 103). In patients with cognitive impairments, which are associated with mental health disorders, the diagnosis of early-onset Alzheimer’s disease may also be considered. Comorbid medical conditions may be particularly important to consider when patients experience a mental disorder after suffering a work-related injury (37). Patients with acute or chronic medical conditions who have high rates of absenteeism or presenteeism, or those who fail to return to work after a physical or psychological injury, should be evaluated for a mood disorder. A Canadian study found higher odds of functional limitations in patients with comorbid mental and physical health disorders, highlighting the importance of treating both mental and physical health to improve functioning and prevent future decline (104).

### 3.3. Discussing work-related mental health disorders with patients

Factors to be considered when conveying a diagnosis of work-related MDD to the patient include information on the diagnosis, the importance of therapeutic alliance, and educational information to address patient concerns, such as stigma/discrimination, loss of employment, isolation, and financial insecurity (37). In helping patients return to work, multiple factors should be considered including weighing various clinical factors, the nature of the work-related stress, the severity of disability and limitations, the motivation of the worker to continue working and the availability of support systems, integrated services, and coordinated treatment plans.

### 3.4. Assessing disability or fitness to work

#### 3.4.1. Factors associated with longer-term inability to work

The Australian guidelines reviewed evidence from seven cross-sectional or cohort studies that assessed patient characteristics and work-related factors that are associated with longer-term inability to work (37). These characteristics shown in Table 6 can help a clinician determine when a person with a mental disorder has the capacity to return to work. Similarly, a prospective observational study of patients on sick leave being treated for mental disorders found that several work characteristics influenced return to work. These included control over decision making, support from colleagues, respect and recognition, and job promotion opportunities (105). Additionally, the person’s self-assessed present and future ability to work and symptom burden also predicted return to work.

**Table 6:** Factors to consider when evaluating a patient’s capacity to work (37).

Patient-related factors	Work-related factors
The severity of mental health disorder Presence of medical comorbidities or sleep disturbance Being male and older Motivation, conscientiousness, and attitude toward work Physical and psychosocial ability to work Personal circumstances (e.g., relationships, finances, housing, physical activity, social/cultural disadvantage)	Workplace environment (e.g., size of the workplace) Ongoing work-related stressors (e.g., conflicts with supervisor or colleagues) Suitability of work (e.g., duties that are appropriate to the patient’s position at work and will not cause stigma)
	Other
	Receipt of appropriate medical treatment Differential diagnosis (e.g., possible malingering)

#### 3.4.2. Definitions of terminology used in assessing fitness to work

From a psychiatric perspective, impairment and disability are substantially different. An individual with a psychiatric impairment does not necessarily have a related work disability. The clinical assessment of work functioning in individuals with mental health conditions is complex and assessment practices are highly variable. The occupational medicine concepts–impairment, capacity, limitations, restrictions, disability, accommodation, and worklessness–can help clinicians organize their approach to the evaluation of the patient’s functional capacity, sick-leave/disability or return to work status (106). Furthermore, understanding and assessing these concepts are critical to evaluating fitness to work, completing insurance forms, and tracking functional outcomes.

##### 3.4.2.1. Impairment

Impairment is what is considered “wrong” with the body’s or, in these cases, the mind’s functioning and is formally defined as “A significant deviation, loss, or loss of use of any body structure or body function in an individual with a health condition, disorder, or disease” (107). Impairment with regard to a psychiatric diagnosis is usually related to the severity of the symptoms, and the degree of functional impairments in various domains such as work, family and social life, and cognition (See Tables 1, 3).

##### 3.4.2.2. Capacity

Capacity in the context of workplace evaluation usually refers to the employee’s current ability or strengths to do their required work. In terms of physical aspects, it includes attributes such as strength, flexibility, and aerobic endurance. In mental health disorders, it includes evaluating mood, anxiety, emotional stability, and cognitive functions such as attention, concentration, and memory among others. Capacity can vary depending on the nature of job demands and context. An important aspect of capacity assessment is understanding job description, expectations, and role of the employee, which is often provided by the employer (see Section 6).

##### 3.4.2.3. Limitations

Functional limitations include the things an individual reasonably cannot do, despite a good effort, as a result of a medical or psychiatric condition. A physical example would be an inability to lift heavy items, due to a fracture or sprain. From a psychological perspective, it may be useful to assess limitations associated with MDD within cognitive, emotional, social, and physical domains (Table 7) (108). Examples of cognitive limitations include problems with memory, concentration, multitasking, completing assignments, meeting deadlines, prioritizing tasks, and reading social cues. Emotionally, patients with MDD often have poor motivation, a low tolerance for stress, social interaction, and getting along with customers or fellow employees. Physically, individuals often have slow response times, trouble sustaining energy and maintaining the stamina to work productively. Clinicians should be mindful of the potential side effects of medications causing workplace limitations due to headaches, gastrointestinal upset, sedation, fatigue, and tremors. It should be emphasized that patients may experience impairment but not have a functional limitation in work role.

**Table 7:** Examples of functional limitations related to mental health disorders.

**Screening out external distractions**	Unable to block out sounds, sights, or odors
**Sustaining concentration**	Restlessness, short attention span, easily distracted, difficulty following instructions
**Maintaining stamina**	Difficulty sustaining energy throughout the work day
**Handling multiple tasks and meeting deadlines**	Difficulty managing assignments, prioritizing tasks, and meeting deadlines
**Interacting with others**	Difficulty cooperating and contributing in group situations
**Fear of authority figures**	Difficulty approaching supervisor or employer
**Responding to negative feedback**	Difficulty understanding and interpreting criticism. Defensiveness due to low self-esteem
**Responding to change**	Difficulty coping with unexpected changes, or tolerating interruptions
**Medication side effects**	Side effects that can affect performance can include: drowsiness, fatigue, dry mouth, blurred vision, tremors, slow response time

Adapted from reference (108).

##### 3.4.2.4. Restrictions

After a thorough medical evaluation, restrictions are activities that clinicians advise against performing as it may cause harm or worsen symptoms. A well-known physical example is the restriction on driving following a diagnosis of epilepsy due to the unpredictable risk of seizure and the danger involved. A psychiatric restriction may apply to someone like a law enforcement officer at risk for suicide who should be temporarily restricted from firearm access. Other examples of restrictions include operating machinery or working in a safety-sensitive position. Temporary restrictions can also apply to potential medication side effects such as sedation, cognitive impairment, and gastrointestinal upset. Restrictions and limitations can be temporary or permanent (Table 7) (108).

##### 3.4.2.5. Disability

Disability can be thought of as an inability to fulfill a role, such as a job, due to an impairment and is formally defined as “Activity limitations and/or participation restrictions in an individual with a health condition, disorder, or disease” (107). A work-related psychiatric disability would be an inability to participate fully in a job role due to the effects of symptoms of the disease on the job demands and subsequent limitations with or without restrictions.

##### 3.4.2.6. Accommodations

An accommodation is an adjustment to the job or work environment that makes it possible for an individual with restrictions or limitations to return to work earlier and safely and re-enable them to perform most of their job duties. For example, an accommodation for an individual with insomnia could be a later start to the workday for a defined period of time or upon reassessment of the response to treatment (see Section 5).

##### 3.4.2.7. Worklessness

More than just losing gainful employment, worklessness speaks about the impact of a loss of meaningful roles in life, such as a worker, student, volunteer, or a parent. The term was introduced in the 2006 British report “Is Work Good for Your Health and Well-Being?” (26). As described above, there is evidence that unemployment is generally harmful to health (25, 26). Avoiding the potential health consequences of worklessness is the foundation of ethical return-to-work/stay-at-work assessments in that it is usually healthier to be at work, in a safe and timely manner.

##### 3.4.2.8. Fitness for duty

The phrase “fitness for duty” is often used in the context of jobs that include a safety-sensitive component, such as police, fire, or railway workers. Safety-sensitivity could include a danger to self, coworkers, the general public, or the environment. When making disability assessments the clinician should be aware that public safety may trump the individual’s right to work (see Section 6) (109).

#### 3.4.3. A practical approach to the assessment of functioning, disability, and fitness to work

The assessment of fitness to remain at work or to consider time off can be challenging. As a clinician, it is important to understand the relationship between mental health symptoms, disorders, and impairments that interfere with carrying out work duties. Primarily, impairments (a deviation or loss of functioning due to a disorder) may or may not lead to a limitation or restriction. It is essential to determine a relationship between how a specific symptom interferes with performance of a specific job requirement. Most existing mental health screening tools, neuropsychological tests, and the mental status examination help in screening for mental health disorders but have limited value in determining performance of work demands or the entire work role. Furthermore, disentangling daily life stressors and work factors can make the fitness assessment challenging.

A fitness assessment should include collateral information about the work environment, reported work stress, conflicts, negative evaluations, and details of the type of work. Functioning assessment will involve an integrative approach using self-report, collateral information from employer and disability professionals, as well as functional and cognitive screening tools such as the SDS, WHO-DAS, or PDQ-5 (see Tables 1, 3). Urine drug screening may be helpful for those with concurrent substance use issues or relapses due to medication non-adherence.

Finally, a formal assessment of barriers to return to work should be identified. Some of the barriers to return to work include the severity of disease, motivation to return to work, work stress, and barriers to mental health services such as recognizing the need for treatment, attitudes associated with stigma, treatment, and a desire to handle the disease independently, and structural issues around finances and access to services.

## 4. Pharmacological and psychological treatments for workplace-related mental health disorders

### 4.1. Pharmacotherapy

Numerous studies, systematic reviews, and meta-analyzes have shown that antidepressants are more efficacious than placebo in adults with MDD (110, 111). Current Canadian guidelines from CANMAT recommend most second-generation antidepressants for the first-line treatment of patients with moderate or severe MDD (Tables 8, 9) (112). Nonpharmacological treatments are preferred for patients with mild MDD.

**Table 8:** 2016 CANMAT guideline recommended first-line antidepressant treatments for MDD in the general population.

SSRIs	SNRIs	Other agents
Citalopram Escitalopram Fluoxetine Fluvoxamine Paroxetine Sertraline	Desvenlafaxine Duloxetine Milnacipran Venlafaxine	Agomelatine Bupropion Mianserin Mirtazapine Vortioxetine

Adapted from reference (112). SNRI, serotonin and noradrenaline reuptake inhibitor; SSRI, selective serotonin reuptake inhibitor. Levomilnacipran and vilazodone were listed second line since data was not available when the guidelines were published, however, they are now generally considered first-line agents.

**Table 9:** 2016 CANMAT guideline recommended antidepressant treatments according to specifiers or dimensions of MDD (112).

Specifiers/dimensions	Recommendations
With cognitive dysfunction	Vortioxetine Bupropion Duloxetine SSRIs Moclobemide
With anxious distress	Use an antidepressant with efficacy in generalized anxiety disorder
With sleep disturbances	Agomelatine Mirtazapine Quetiapine Trazodone
With somatic symptoms	Duloxetine (pain) Other SNRIs (pain) Bupropion (fatigue) SSRIs (fatigue) Duloxetine (energy)

Adapted from reference (112). SNRI, serotonin and noradrenaline reuptake inhibitor; SSRI, selective serotonin reuptake inhibitor.

An important caveat is that existing guidelines and recommendations for pharmacological and nonpharmacological approaches include data drawn from clinical trials, which generally do not include comorbid populations and, therefore, differ substantially from the patients seen in real-world clinical practice.

### 4.2. Targeting pharmacotherapy to work-related MDD

When evaluating therapies for work-related MDD, it is important to assess antidepressants beyond their effect on depressive symptoms and to consider other outcomes such as cognition and reward processing, work function, sick leave, and return to work.

#### 4.2.1. Impact of antidepressant treatment on cognitive function

As discussed in Section 3, impairments in cognition play a major role in workplace dysfunction. Cognitive impairment has been associated with decrements in social, occupational, and global functioning, and has been shown to have a greater impact on workplace dysfunction than the severity of depressive symptoms (51–55, 57).

A meta-analysis of 9 placebo-controlled RCTs, which included mainly vortioxetine, duloxetine, and SSRIs, found that antidepressants had a positive effect on psychomotor speed and delayed recall (113). Canadian guidelines include suggestions for specific pharmacological treatments based on clinical specifiers and dimensions of MDD (112). For patients with cognitive dysfunction preferred pharmacotherapies include: vortioxetine (level 1), bupropion, duloxetine, or an SSRI (level 2), or moclobemide (level 3) (112). Nonetheless, the issue of generalizability of the clinical trial data to work-related MDD is lacking.

#### 4.2.2. Impact of antidepressant treatment on work functioning

As described in Section 3, measurement tools to assess functioning can be very useful during clinical assessment. One of the most commonly used measures of work-related functional outcomes is the Sheehan Disability Scale (SDS), and in particular the SDS-work item (i.e., the symptoms have disrupted your work/school work; rated on a scale of 0–10) (72). One limitation of the SDS is that it captures role functioning at work rather than work functioning. Although other tools such as Work Limitations Questionnaire (WLQ) (75) and Work Productivity and Activity Impairment (WPAI) (76) capture work functioning, they are not routinely used by most clinicians as they were developed for group-level usage.

Antidepressant treatment trials with work-related outcomes were identified from systematic reviews (114–117), updated with Medline searches as of February 2023. A total of 27 RCTs were identified that provided subjective measures of work-related disability and/or productivity (mainly SDS-work measures). Functional outcomes are generally secondary endpoints in RCTs and often the data are not included in the primary study publication. For this review, studies were included if they were randomized, double-blind, placebo- or active comparator-controlled study design, included a work functioning outcome measured at baseline and endpoint, and the data were available in the primary publication or one of the systematic reviews. The results of 22 studies comparing an antidepressant to placebo (118–139), and 5 studies comparing 2 different antidepressants (140–144) are shown in Supplementary material S4. Most selective serotonin reuptake inhibitors (SSRI), serotonin and noradrenaline reuptake inhibitors (SNRI), noradrenaline and dopamine reuptake inhibitors (NDRI), and multi-modal antidepressants showed positive benefits on measures of work impairment; however, results of individual studies were highly variable. This may be due in part to the fact that work outcomes were secondary endpoints, and studies may not have been powered to adequately assess these outcomes.

In summary, the body of evidence suggests that antidepressants from different classes are associated with secondary improvements in work functioning, but there is little evidence to suggest a specific medication is preferable for patients with work-related MDD.

#### 4.2.3. Impact of antidepressant treatment on sick leave/return to work

A systematic review of interventions to improve return to work in patients with MDD identified 6 studies assessing the effects of antidepressants on the duration of sickness absence (Table 10) (41, 145–150). The results of 3 studies comparing SSRI to SNRI were inconsistent (41, 145–147), with 2 studies showing no differences between agents (145, 146), and one showing a greater benefit with the SSRI, escitalopram compared to the SNRI, duloxetine (147). Another study also showed a benefit with escitalopram compared to another SSRI, citalopram (148). No differences were found between a SSRI and a TCA in reducing sickness absence or work functioning as measured by the Social Adjustment Scale (SAS) work composite (149). Compared to placebo, one study found no benefit of a TCA or a MAO on sickness absence, but a significant positive effect on the work (150).

**Table 10:** Randomized controlled trials measuring the effects of antidepressants on sick leave (41).

Variable	Agents	Days of sickness absence, SMD SMD between groups (95% CI)
**SSRI** ***vs*** **SNRI**		
Fernandez et al. 2005 (145)	Escitalopram vs. venlafaxine XR	−0.03 (−0.37, 0.31)
Romeo et al. 2004 (146)	Paroxetine vs. mirtazapine	0.28 (−0.13, 0.69)
Wade et al. 2008 (147)	Escitalopram vs. duloxetine	−0.57 (−0.88, −0.26)
**SSRI** ***vs*** **SSRI**		
Fantino et al. 2007 (148)	Escitalopram vs. citalopram	−0.31 (−0.54, −0.07)
**SSRI** ***vs*** **TCA**		
Miller et al. 1998 (149)	Sertraline vs. imipramine	0.08 (−0.08, 0.25)
**Any antidepressant** ***vs*** **PBO**		
Agosti et al. 1991 (150)	TCA or MAOI vs. placebo	0.48 (−0.05, 1.00)

CI, confidence interval; SMD, standardized mean difference.

Two other studies looked at the number of underproductive or lost days per week as measured by the SDS subscores (118, 143). One study found decreases with both vortioxetine (from 5.3 to 2.7 days) and venlafaxine XR (from 5.2 to 3.0 days) at week 8, but between-group statistics were not reported (143). In another study, reductions were significantly greater with agomelatine compared to placebo (number of lost work days −1.13 days, and number of underproductive days −2.03) (118). In summary, antidepressants seem to reduce sick leave and earlier return to work, but no differences were identified between classes.

#### 4.2.4. Impact of pharmacotherapy on work outcomes in real-world studies

The benefits of antidepressant treatment on work functioning, including absenteeism and presenteeism, have also been demonstrated in observational studies conducted in real-world settings.

The Prospective Epidemiological Research on Functioning Outcomes Related to Major depressive disorder (PERFORM) study was a large 2-year European observational study (151). Initiation (79% of patients) or switch of antidepressant monotherapy (21%) (per routine clinical practice) was associated with improvements in both depressive symptoms (e.g., PDQ-5, PHQ-9 scores) and functional impairment (e.g., SDS total score, WPAI absenteeism and presenteeism scores) (151).

The Assessment in Work productivity and the Relationship with Cognitive symptoms (AtWoRC) study was a real-world open-label study in Canadian working adults with MDD. The study showed significant improvements in cognitive function and workplace productivity with vortioxetine at both week 12 (58), and 1 year (68). WPAI presenteeism was more strongly correlated with other measures of workplace productivity (i.e., SDS work/school and the overall WLQ productivity loss score) than WPAI absenteeism (152). Results of a pharmacoeconomic analysis showed that these improvements were associated with a savings of $4,550/per patient/per week (2017 CAN$) over the 1-year study (33). In the Real-Life Effectiveness of Vortioxetine in Depression (RELIEVE) study, vortioxetine significantly improved all work productivity measures (sick leave, absenteeism, and presenteeism) compared to baseline at week 24 (153).

Recently there has been interest in the use of esketamine and ketamine, with CANMAT recommending ketamine as a third-line choice for patients with treatment resistant depression (154). In terms of effects on functioning, a meta-analysis of randomized and observation studies found mixed results were reported with respect to effects on general functioning (155). Data from the Canadian Rapid Treatment Center of Excellence on the use of ketamine in 171 patients with treatment resistant depression in community practice found a significant reduction in workplace disability, and symptoms of presenteeism and absenteeism (156).

### 4.3. Psychotherapeutic strategies

In the general population, RCTs, systematic reviews, and meta-analyzes, have shown that psychotherapies, particularly CBT, are effective for the treatment of MDD compared to placebo (157–161).

Canadian guidelines from CANMAT recommend CBT, interpersonal therapy (IPT), and behavioral activation (BA) for the first-line treatment of acute MDD (Table 11) (162). Combination therapy with antidepressants is preferred as this has generally been shown to be more effective than either treatment alone, particularly for patients with moderate to severe depression (25, 26, 160, 161).

**Table 11:** Psychological therapies for acute treatment of MDD (162).

Recommendation	Psychological treatment
1st line	Cognitive-behavioral therapy (CBT) Interpersonal therapy (IPT) Behavioral activation (BA)
2nd line	Mindfulness-based cognitive therapy (MBCT) Cognitive-behavioral analysis system of psychotherapy (CBASP) Problem-solving therapy (PST) Short-term psychodynamic psychotherapy (STPP) Telephone-delivered CBT, IPT Internet- and computer-assisted therapy
3rd line	Long-term psychodynamic psychotherapy (PDT) Acceptance and commitment therapy (ACT) Video-conferenced psychotherapy Motivational interviewing (MI)

### 4.4. Targeting psychotherapy for work-related MDD

#### 4.4.1. Impact of psychotherapy on functioning

A meta-analysis of RCTs found that both psychotherapy and pharmacotherapy had significant beneficial effects on functioning (SDS or SAS) and quality of life (Q-LES-Q or SF-36) in patients with depression (163). The combination was more effective than either treatment alone.

#### 4.4.2. Impact of psychotherapy on work functioning

In a meta-analysis of 28 studies of the effectiveness of workplace-delivered digital mHealth interventions (e.g., CBT, mindfulness/meditation, stress management) there was a small, but significant effect on engagement and productivity, but effects on absenteeism and presentism were not significant (164). In a pragmatic trial, a digital mindfulness app showed similar benefits; however, drop-out rates were very high with only 15% of participants completing all 5 assessments over 8 weeks (165). An analysis of studies over time found no evidence of an increase in efficacy of these interventions over the past decade, despite a tripling of the number of studies and the advances in technology (166). Of note, most of these studies were on unselected employees, and only a minority were on individuals with mental health conditions or high stress levels (164–166).

A pilot study showed a significant improvement in work productivity associated with a brief group CBT intervention that was tailored to work-related issues (167).

#### 4.4.3. Impact of psychotherapy on sick leave/return to work

In terms of specific work-related outcomes, a systematic review and meta-analysis found 16 RCTs that assessed the efficacy of psychotherapies for return to work in patients on sickness absence due to mental disorders (depression, stress disorders, and mixed diagnoses) (168) Among patients with mental disorders, the psychological interventions were significantly more effective in reducing sickness absence overall, and compared to treatment as usual (effect sizes = 0.13) (Table 12). There were no significant differences between different psychological treatments (0.21) or psychological vs. non-psychological treatments (0.37). In addition, studies that included an intervention that targeted work-related processes (9 of 30 trials) were more effective than those that did not (Table 13) (168). In these studies, the types of return to work interventions varied substantially, and the key mechanisms to target in work-focused interventions remain unclear.

**Table 12:** Effect sizes (odds ratios) for proportions of patients with mental disorders with partial or full return to work (168).

Comparison	OR (95% CI)
All psychotherapy studies	1.67 (1.15–2.41)
Psychotherapy vs. other psychotherapy	2.46 (0.99–6.10)
Psychotherapy vs. non-psychotherapy	0.89 (0.43–1.84)
Psychotherapy vs. TAU	1.54 (1.02–2.31)

CI, confidence interval; OR, odds ratio; TAU, treatment as usual.

**Table 13:** Effect sizes (odds ratios) for proportions of patients with mental disorders with partial or full return to work according to the type of psychotherapy (168)

Variable	OR (95% CI)
**Treatment type**	
Cognitive behavioral therapy (CBT)	1.586 (0.95–2.66)
Work-CBT (W-CBT)	1.969 (0.71–5.45)
Multi-modal CBT (MMCBT)	0.902 (0.62–1.31)
Problem-solving therapy (PST)	1.403 (0.92–2.15)
**Format**	
Group	1.078 (0.67–1.74)
Individual	1.410 (0.99–2.00)
Group + individual	2.090 (0.60–7.25)
**Workplace intervention included**	
No	1.099 (0.83–1.45)
Yes	2.548 (1.35–4.83)

CI, confidence interval.

Similarly, a meta-analysis of 45 RCTs, found small but significant reductions in sick leave and symptoms with psychological treatments in patients with common mental disorders (e.g., diagnosis or symptoms of depression, anxiety, stress or insomnia) (169). There were no significant differences between CBT, collaborative care, work-focused interventions, or problem-solving therapy. An analysis that included 21 RCTs found that web-based psychological interventions delivered in the workplace had significant effects on both psychological well-being and work effectiveness compared with control groups (170). In this meta-analysis, no significant differences were found between CBT versus other psychological therapies, and offering guidance versus self-guidance for the work effectiveness outcome. In another meta-analysis of 6 studies internet-delivered CBT was not associated with a reduction in sickness absence compared to control groups (171).

## 5. Integrative treatment strategies for work-related mental health disorders: a collaborative approach

### 5.1. The role of clinicians in facilitating full recovery and return to work

**Figure 1:**
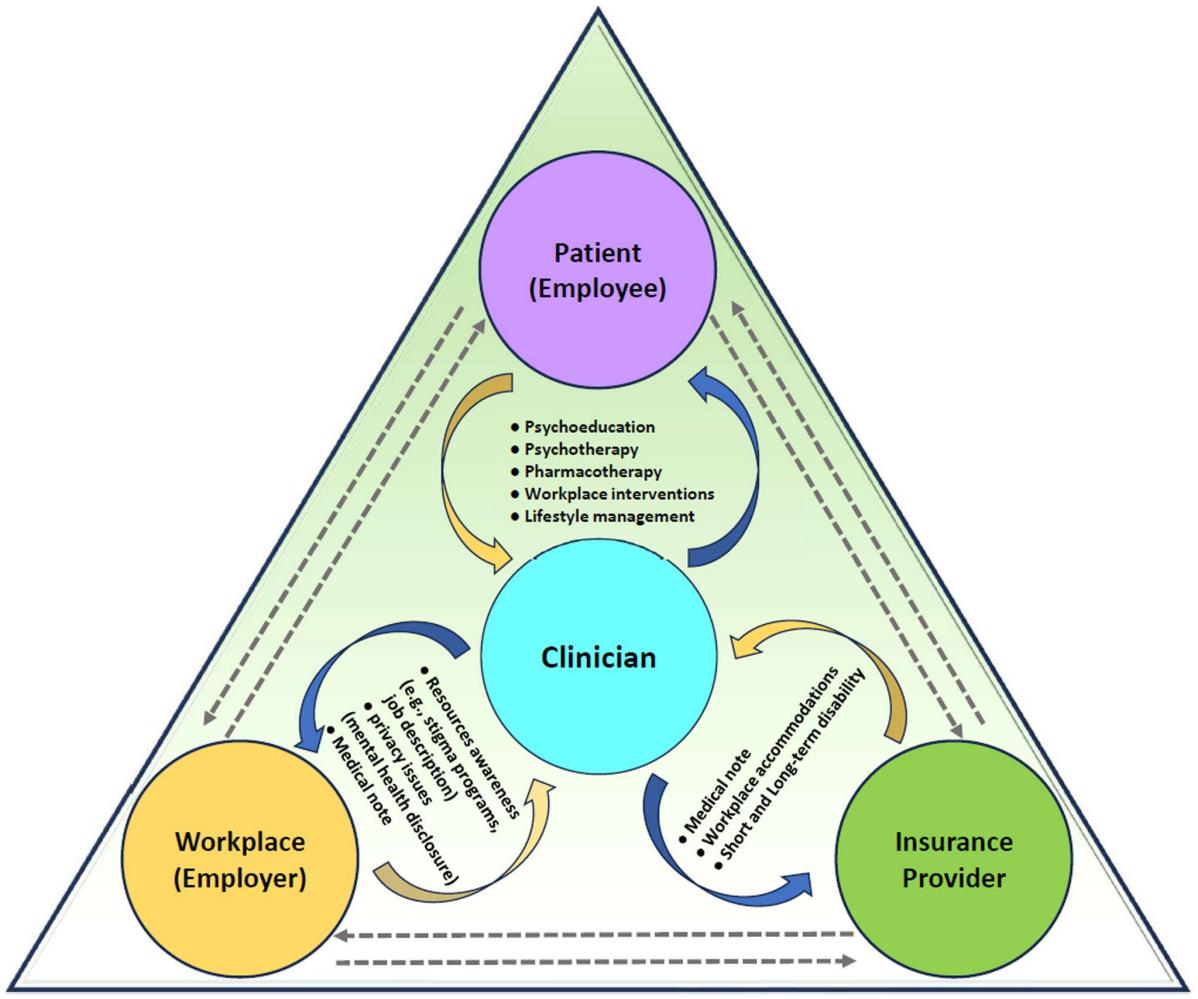
Elements of integrated care for patients with work-related mental health disorders.

Clinicians managing patients with work-related depression have multiple roles including patient care, as well as integrating with the workplace (e.g., human resources) and insurance provider (Figure 1) (37). Factors in the work environment that impact return to work were discussed in Section 2 (16, 37, 105). Although physicians may not be able to directly impact factors at work, they should be aware of the challenges their patients might be facing, and any workplace programs that are available to their patients. The need for sick leave, workplace accommodations, or referral to other specialties (e.g., occupational medicine specialist, occupational therapy, psychology, physiotherapy, or patient advocacy groups), may be mediated in part by the resources currently available at the patient’s workplace. As a part of integrative treatment strategies, the sections below will address the role of the clinician with the patient (employee), workplace (employer), and insurance provider.

### 5.2. The role of clinician with the patient

#### 5.2.1. Psychoeducation, pharmacotherapy, and psychotherapy

For patients with work-related mental disorders, effective interventions include psychoeducation, pharmacotherapy, and psychotherapy (see Section 4), and work-focused interventions (e.g., addressing interpersonal conflicts, providing work accommodations) (Table 14) (16, 172). A systematic review including 36 studies found that multi-domain interventions that included at least 2 of 3 domains (e.g., healthcare provision, service coordination, and work accommodation) significantly reduced duration away from work and had a positive impact on cost outcomes among patients with mental disorders (173). For example, the addition of psychological treatment, such as online CBT, to usual care reduced sick leave by 15 days (41). Other interventions, such as improving or streamlining the organization of care, adding a structured telephone outreach and care management program to pharmacotherapy, or adding specific providers for depression care, also helped to reduce sickness absence days (41).

**Table 14:** Key elements in multi-domain interventions (16, 172).

Elements	Description
Screening	Early identification through privacy-protected tools, such as web-based programs
Psychoeducation	Empowers employees by reducing stigma and increasing awareness of treatment options
Antidepressants	Medication as needed, generally through primary care provider. Patients who respond to medication often show improved work outcomes (see Section 4)
Work-focused CBT	CBT programs tailored to the work environment appear to be more effective than general CBT programs (see Section 4)
Care management	Counseling (e.g., through Employee Assistance Programs) may not be adequate alone, but maybe useful in combination with other strategies
Work coaching and modification	Workplace accommodations (e.g., modified tasks, or reduced working hours)
Engagement	Workplace outreach strategies such as contacting employees who screen positive for depression, or motivational interviewing
Coordination of care	Integration/coordination with prescribers and other providers
Measurement-based care	Clinicians and employees track depression symptoms and work performance over time to tailor management to ensure full recovery
Shared decision-making	Clinicians provide evidence and support to help patients make informed choices, which can increase patient satisfaction, treatment adherence, and effectiveness

CBT, cognitive-behavioral therapy.

#### 5.2.2. Workplace interventions

Clinicians alone cannot affect all the dimensions needed to ensure a patient’s functional recovery and return to fully productive work. In addition to clinical care, clinicians must be aware of possible workplace interventions as this has been shown to improve outcomes in work-related depression. A meta-analysis of nine studies in patients with MDD found that a combination of a work-focused intervention and a clinical intervention reduced sickness absences by 25 days within the first year of follow-up, in addition to reducing depressive symptoms (41). Work-focused interventions assisted employees in getting changes in work tasks or working hours, supported a gradual return to work, or helped workers adjust to work situations (41).

#### 5.2.3. Lifestyle management

In addition to pharmacotherapy, psychotherapy and workplace interventions, lifestyle management approaches such as exercise should be strongly considered to manage workplace depression. While many studies have assessed the use of exercise-based interventions for mental disorders, specific evidence supporting the use of such modalities for improving work-related outcomes is scarce. A Cochrane systematic review of interventions to improve return to work in patients with MDD included two randomized controlled trials (41, 174, 175). For the outcome of reducing sickness absence, supervised strength exercise was more effective than relaxation, whereas aerobic exercise was not more effective than relaxation or stretching. Two additional randomized trials were found (176, 177). In a 12-week trial among 946 patients with depression, physical exercise was as effective as usual care by a physician in improving self-rated work capacity (176). Another study found that while both treatments lowered the rates of long-term sick leave over time, physical exercise was significantly superior to treatment as usual at 3 months, but not at 1 year (177).

### 5.3. The role of clinician with the workplace

#### 5.3.1. Engage with the workplace stakeholders

In treating work-related depression clinicians are encouraged to engage with the workplace to enhance treatment outcomes. Unfortunately, multidisciplinary collaboration for the management of patients with work-related mental disorders may be under-used in clinical practice (178). In one survey, 40% of primary care physicians had never had contact with an occupational health physician, and 49% had not had contact with psychotherapists (179).

Clinicians should liaise with workplace coordinators (with the permission of the patient). Some companies (especially larger organizations) have dedicated personnel to manage disability claims and coordinate care, whereas others may offer these services through their insurance companies. A scoping review found that the lack of coordination between stakeholders involved in the process creates confusion around returning to work for patients with a mental disorder. A coordinator can help involve all relevant stakeholders (178). The coordinator should be knowledgeable about rehabilitation and mental health services, organizational procedures and management values, and the needs of workers and the organizations (178). Liaising with these coordinators (with permission of the patient) can help ensure access to comprehensive treatment including medication and psychotherapy, facilitate communication with patients on sick leave, monitor progress, and develop a plan for returning to work. These coordinators have the added benefit of helping to ensure that patients do not feel cut-off and isolated, especially if they have limited social support.

#### 5.3.2. Clinician responsibilities and privacy issues when communicating with a patient’s workplace

Coordination of care between the various stakeholders is vital to promote successful return to work. With the patient’s consent, clinicians are obliged to communicate with other care providers, the patient’s employer, or other individuals or entities involved to facilitate the return to work process and prevent the fragmentation of care. Often, it may be helpful to have the patient participate in these conversations. An additional benefit of constructive communication between the clinician and a patient’s employer is the identification of workplace factors, both medical and non-medical, hindering worker recovery. Such a collaborative approach provides a greater opportunity to address potential barriers (37).

Achieving successful return to work has some essential ingredients, regardless of the health condition. Physicians are just part of the recipe. The Institute for Work and Health has developed a booklet called “Seven principles for successful return to work”^2^ which can help clinicians understand the process (180).

Even when a patient provides consent for a clinician to communicate with third-parties, an employer is not entitled to know the specifics of a worker’s mental health diagnosis. However, an employer may request that a clinician confirm that the employee’s condition limits or restricts them from performing the essential duties of their role. If they are unable to work in any capacity, then medical documentation would need to meet thresholds for “disability” as defined by an insurance provider.

When discussing a work-related mental disorder with a patient’s employer or providing information to other relevant third parties such as a private insurer, it is the clinician’s responsibility to ensure that the focus is on the workplace and the employee’s needs and functional capacities as they relate to the work role. Clinicians may find it helpful to obtain a detailed job description, or checklist describing the duties and expectations of the job. The Canadian Medical Association has published a policy statement outlining the role of clinicians in the completion of third-party forms (Table 15) (181).

**Table 15:** Summary of Canadian Medical Association policy statement on third-party forms (181).

Clinician’s role	Patient’s role
Provide objective information on physical/psychological impairments and abilities, limitations/restrictions, and time frames/prognosis for purpose of administering program or benefit Ensure form completed accurately and objectively according to provincial regulatory agency Focus on medically indicated restrictions when asked to provide opinion on functional abilities of patient Secure patient consent to disclose information to any 3rd-party	Review 3rd-party forms and be aware of info being requested If applicable, complete sections requesting information on subjective complaints and self-reported function Be aware that their consent authorizes clinicians to accurately and objectively explain condition to 3rd-party

#### 5.3.3. Helping patients communicate with workplace personnel about their illness

The individual worker must decide whether, and to what extent, to discuss their mental disorder with their supervisor or other relevant individuals at the workplace. There are situations where such a discussion would be beneficial or even necessary to provide the required accommodation and support. The worker should be encouraged to engage their supervisor as support, as this will facilitate an understanding of changes in work behavior, and a discussion of the eventual resumption of usual productivity levels. It also should be noted that there are instances in which workers may decline to disclose their disability to their employer and continue to be productive at work. The decision to discuss their mental disorder with a supervisor should be made while considering the type of job and culture of the workplace.

Although workers are legally entitled to accommodation, a knowledge and skills gap may exist among employers regarding their awareness of the legal expectations, and effective approaches regarding how to discuss and provide needed accommodations for a worker. Similarly, workers also may not know how to articulate the difficulties they are experiencing with their job assignments, or not know how to communicate their needs. Patients should be reminded that it is their responsibility to inform their employer about specific needs as they arise and be prepared to provide supporting medical documentation if requested.

In the 4-part series, *Healthy Brains at Work*, conducted by the Conference Board of Canada, 93% of employers surveyed had supportive policies and practices in place to provide the flexibility needed for workers to remain on the job while receiving treatment or recovering (182).

The decision of whether to inform co-workers about a mental health issue remains entirely with the patient. There is a greater potential for a more supportive working environment (105, 183, 184). Supervisor and colleague support have been identified as important factors in predicting return to work among patients with mental disorders (105, 183, 184).

Factors that might influence a worker’s decision to disclose their mental health disorder include: receiving advice from a trusted person (e.g., a physician, a colleague, or a personal contact), managerial changes (e.g., new policy, the manager begins talking about mental health issues, or the manager is replaced), and experiences of colleagues (seeing others have positive response among others at work) (185, 186).

#### 5.3.4. Rehabilitation services awareness

Clinicians must be aware of the type and quality of rehabilitation services as an important factor in promoting return to work (37). A study of two vocational approaches found that the most important factors predicting return to work were: feelings of hope and self-esteem/efficacy among patients, positive attitudes and behaviors among professionals (e.g., genuine interest, engagement, and support), and integration of both health and vocational services in return to work programs (187). Meta-analyzes and RCTs show that interventions by occupational health services (generally problem-solving treatment or CBT) can reduce the number of sick days compared to usual care among patients with work-related mental disorders (187). However, these interventions did not consistently improve symptoms of mental disorders, underscoring the need to simultaneously target both return to work and improvement of symptoms with an integrated approach.

#### 5.3.5. Stigma

As discussed in Section 1, stigma regarding mental health disorders continues to exist in the workplace. In this case, there are a number of things to consider in the disclosure decision (1): who to disclose to (2), timing (3), message content, and (4) communication style (188). In workplaces in which stigma exists, patients may have concerns around perceived or actual negative effects on career opportunities, or non-supportive environments (185, 186). As a clinician, be prepared to challenge and help the patient to work through possible distortions associated with mental health disorders such as feelings of persecution, personalization, and “catastrophizing.” Ultimately, self-stigmatization may be a greater barrier to accessing the needed support to achieve a successful return to work. However, after these discussions, clinicians should be accepting of the patient’s perceptions of their individual workplace and support their decision.

Clinicians should also be aware of the potential impact of stigma in the workplace on patient recovery from a work-related mental disorder, particularly MDD. Stigmatizing attitudes in employers, refusal to implement accommodations, as well as pressure toward productivity and performance, have been documented as negatively influencing the return to work of employees with depression (189, 190).

A systematic review of 16 studies found that anti-stigma interventions at the workplace can improve employee knowledge and supportive behaviors toward people with mental disorders (191). Workplace awareness and stigma reduction programs have also been shown to encourage employees to seek treatment for mental health disorders (192).

In Canada, educational workplace initiatives such as The Working Mind program developed by the Mental Health Commission of Canada are effective in reducing stigma and increasing resilience and coping abilities, across a variety of settings (193), and benefits were maintained at a 3-month follow-up (194). These workplace programs include strategies to reduce stigma, improve awareness of the symptoms of mental disorders, promote coping skills, and provide information about workplace policies and practices to help employees who experience problems (193).

### 5.4. The role of clinician with the insurance provider

As a part of the management of work-related depression clinicians must collaborate with the insurance provider with regular and updated information. The amount of required documentation of the patient’s disease progress, limitations, restrictions, prognosis, and time to return to work can be overwhelming and frustrating to clinicians as they navigate through the disability process. However, knowledge on the medical note, assessing short and long-term disability, and workplace accommodations can help alleviate the clinician’s burden.

#### 5.4.1. Medical note

In completing the medical note (sick note), clinicians should be aware that the Canadian Medical Association (CMA) policy position is that sick notes are an unnecessary burden for clinicians and that employers should be restricted from requiring an employee to provide a sick note for short leaves of absence due to personal illness, injury, or medical emergency (195). However, whether employers can require clinicians’ confirmation of the employee’s inability to go to work varies widely. Some provinces have legislation pending addressing the issue of sick notes and some individual employers have introduced policies abandoning sick notes for short-term absences. Reporting to the Workers’ Compensation Boards in Canada may be necessary.

As described in section 1, sustained unemployment has many negative consequences. For individuals in jobs with a safety-sensitive component (e.g., emergency services, heavy equipment operators, pilots, transportation workers), return to work assessments are more comprehensive and focused on limiting risk. As a guiding principle in fitness for duty assessments, public safety may trump the individual’s right to work. The policy of the CMA states: “The treating physician must be aware of the risks to the patient, his or her coworkers or the public that could arise from the patient’s condition or drug therapy. If the patient’s medical condition and the nature of the work performed are likely to endanger the safety of others significantly, the physician must put the public interest before that of the patient/employee” (109).

In order to return to safety-sensitive work after a period of illness the worker must demonstrate normal or adequate capacity to reliably perform and respond to safety-sensitive demands. For example, as described in the Canadian railway medical guides (196), “The individual’s bipolar I disorder has been in remission for a continuous period of 1 year during which the individual has been maintained on a stable dose of medication.” In essence, there must be no imminent risk of relapse of the mental health disorder.

Finally, if a medical code is requested, provide ICD-11 or DMS-5 codes, rather than non-descript terms such as “mental health” or “stress.” Consider the sick note as a medical intervention with associated clinical benefits and risks.

#### 5.4.2. Workplace accommodations

Clinician should facilitate setting up work accommodations to help address functional limitations in patients with mental disorders, and improve their ability to successfully complete work-related tasks (Table 16) (30). Occupational health and human resources are the first points of contact when making accommodation requests and submitting relevant medical forms. They can ensure that any restrictions and limitations are communicated to the manager, without conveying diagnosis or other sensitive medical information, so that appropriate accommodation can be implemented and monitored. Using booklets such as “Supporting Employee Success”^3^ can be helpful in understanding the process (180).

**Table 16:** Types of workplace accommodations to address functional limitations in patients with mental disorders (30).

Category	Types	
Communication	Job coaching Task planning and setting priorities Providing feedback and instructions Weekly/regular meetings with supervisor	Extended job training Employee Assistance Program service Training/orientation for supervisor and colleagues
Scheduling	Flexible or part-time work schedule Extra time to complete tasks	Slowing pace of tasks
Job description	Gradual task introduction Constant job description over time Job sharing/trading	Postponing tasks Work from home Change to a different position
Physical space	Access to water, rest/private area Changes in spatial arrangements	Changes to lighting arrangements Lower noise levels

A systematic review of 15 studies found that work accommodations reduced limitations in the work place, improved length of job tenure, and reduced the severity of some mental disorders (30). Work accommodations have been shown to increase job tenure by between 7 to 24 months compared to individuals receiving no accommodations (197, 198). A study of 715 Canadian employees with a current mental disorder found that receiving needed work accommodations was associated with a 25% lower risk of having a mood/anxiety disorder during the 1-year follow-up (32). Programs such as job coaching are associated with minimal costs for both start-up and maintenance, and employee assistance programs have minimal direct and indirect costs (30, 199).

Failing to disclose a workplace-related mental disorder can limit access to accommodations (30, 198, 200). It is important to educate patients on the importance and benefits of workplace accommodations, and patients may need to talk to their employer about specific accommodations that could be helpful (Table 16).

Assessing accommodations is a collaborative effort between the clinician, patient, and workplace. Clinicians should obtain consent from the patient prior to communicating with other stakeholders to better understand job description and role, to help determine possible accommodations such as reduced work hours or workload, re-evaluate job responsibilities, or a possible transfer to a new role. Accommodation can be temporary or permanent in nature and should be re-evaluated periodically to monitor and evaluate treatment effects on patient outcomes.

Furthermore, the Work Accommodation and Natural Support Scale (WANSS) has been developed and validated for people with mental illness returning to work (200). Two versions are available for implementing accommodations in the workplace, which consider the needs for employees and the feasibility for managers/supervisors (201–203). The tools described in Table 3 can also be useful to monitor the effects of MDD on functioning and work productivity, and may be helpful when assessing the need for workplace accommodations.

#### 5.4.3. Short and long term disability

If a patient is unable to return to work after the sick leave, the clinician must assess if the patient can return to work with or without accommodation or be eligible for disability. Short-term disability refers to a limited period of time that qualifies a person for benefits. Depending on the insurance provider the disability benefits and their definition of short-term may vary. In Canada, most short-term disability benefits are between 3 and 6 months before transitioning into long-term disability.

Charts to track recovery can also be very helpful, such as those in the booklet “Supporting Employee Success,” or those published in the “Global Business and Economic Roundtable on Mental Health”^4^ (1). These charts can help clinicians track the patient’s symptoms and functioning, and their ability to perform job duties, and can be useful when completing insurance forms. It should be noted that a decrease in symptoms may not necessarily be accompanied by an increase in work functioning.

### 5.5. Factors associated with recovery and sustainable return to work

Clinicians should be aware of a number of patient, treatment, and workplace factors that may adversely affect the management of work-related MDD (Table 17) (37). These data were identified in a systematic review of studies assessing factors that predict return to work in patients with mental disorders conducted for the Australian guidelines (37). The review identified primarily observational, cohort studies, but included over 300,000 patients, and the findings were very consistent. Patient-related factors include high stress levels unrelated to work, poor treatment adherence, and poor attitudes toward returning to work (Table 17) (37). Poor health prior to sick leave, duration and severity of the mental disorder, physical injury, and poor health behaviors (e.g., smoking, substance use, over or under weight) should also be considered in patients having trouble returning to work. Finally, factors in the work environment such as harassment, stress, and poor communication play a role (see Section 3) (16, 37, 70, 71, 105). Furthermore, there are gaps in evidence such as the relative lack of data on marginalized groups and low-wage workers, which will impact recovery and return to work.

**Table 17:** Factors that might impair the management of workplace-related MDD (37).

Patient-related factors	Medical factors	Employment/ workplace factors
Age > 40y Stressful life factors independent of work Poor treatment adherence Perceived injustice Attitudes toward returning to work ↓Expectations regarding ability to return to work	Persistent symptoms prior to sick leave ↑Severity of mental health condition Comorbid smoking, alcohol, or drug use ↑Duration of symptoms and sick leave at baseline Extensive physical injury Chronic pain Overweight/underweight Rehabilitation service quality	Job/work stress Poor communication with supervisor/ employer Harassment and bullying prior to mental health condition

In other systematic reviews, the most important predictive factors for sickness absence among workers with a mental disorder were previous mental disorders, greater symptom severity, previous absenteeism, comorbidity, high job demands, low job control, high job strain, female gender, lower educational level, smoking, and low perceived general health (204, 205). Factors predicting return to work included perceived supervisor- and colleague-support, work accommodations, positive attitudes, self-efficacy, young age, and higher education levels (184, 205, 206). In addition, delay in initiation of antidepressant therapy after diagnosis of MDD has been associated with longer duration of sick leave, including a 6-fold higher risk of having more than 30 sick days (207).

In addition, clinicians should be aware that low social support is an independent predictor of poorer mental health and lower work ability compared to high social support (51–53). In addition, supervisor, colleague, and non-work-related social support have an impact on workplace bullying, mental distress, and sickness absence (183). Physicians should recognize the importance of identifying people who have limited access to social support and refer them to advocacy or other groups.

## 6. The role of clinician as a patient advocate

### 6.1. Helping patients understand their rights relating to workplace-related illness

Canadian employers have a legal obligation to maintain a physically and psychologically healthy and safe work environment (208). Although some patients may be aware of their rights, it is useful for clinicians to remind patients that employers cannot discriminate based on the presence of any illness, including addictions, and that there is a legal right to accommodation in Canada (16, 208, 209). In fact, the Canadian Labor Code states that “no employer shall dismiss, suspend, lay off, demote or discipline an employee because of absence from work due to work-related illness or injury” (209). While Canadian labor law is clear regarding a worker’s right to appropriate accommodation, it is not incumbent upon the employer to solicit information from the employee as to their specific needs (See Section 4 for a discussion of workplace accommodations).

Employers have a duty to inquire when they are concerned about health-related impairment. They should recommend the worker seek help but ultimately the onus is on the worker to do so. In some cases, workers may need legal assistance to exercise their rights.

It is the responsibility of the employee to inform their employer of their need for accommodation, and to participate in finding solutions. Workers (patients) need to be flexible if their employer suggests alternative solutions that may work better for the employer while still meeting the employee’s needs. If a worker is reluctant to disclose their need for accommodations, they may need help identifying someone at work who they trust and who is in a position to give work-related advice. This work ally can help the employee in determining how much to disclose, what accommodations they can ask for, and how to ask for them. When the worker decides to disclose their need for accommodations, it is important that identifying and implementing workplace accommodations be done collaboratively between employer and employee (210), and be reviewed over time depending on changes in the course of the worker’s condition. Most accommodation is temporary but may become permanent if further improvement is not expected with the worker’s condition.

Using booklets such as “Supporting Employee Success” (see text footnote 3) can be helpful in understanding the process (180). Charts to track recovery can also be very helpful, such as those in the booklet.

### 6.2. Helping patients navigate government programs/resources and complete disability forms

Identifying relevant programs and navigating complex forms from federal or provincial government agencies can be challenging in the best of circumstances and seemingly insurmountable for patients with work-related mental disorders.

The resource lists provided in Tables 18–20 can be useful resources for clinicians to discuss with their patients. Table 18 provides resources to help patients better understand their condition and identify support services. Table 19 lists useful resources for those needing help communicating with their supervisor, the human resources department, or work colleagues about their mental health issues. Finally, useful websites for disability or benefit-related information are provided in Table 20.

**Table 18:** Resources for patients on mental health disorders and social support programs.

Organization	Link
Canadian Center for Occupational Health and Safety (CCOHS)	http://www.ccohs.ca
CCOHS: Mental Health	https://www.ccohs.ca/topics/wellness/mentalhealth
Healthy Minds at Work	https://www.ccohs.ca/healthyminds
Canadian Mental Health Association (CMHA)	http://www.cmha.ca
CMHA: Resources for Accommodation and Accessibility	https://ontario.cmha.ca/documents/accommodation-and-accessibility
Mental Health First Aid Canada	https://www.mhfa.ca

**Table 19:** Resources for patients on communicating with their workplace.

Organization	Link
CCOHS Fact Sheet: Courageous Conversation (tips for employers when discussing mental health concerns)	https://www.ccohs.ca/oshanswers/psychosocial/mentalhealth_conversations.html
Mental Health Commission of Canada (MHCC) (innovative programs to support mental health/wellness)	https://www.mentalhealthcommission.ca/English/what-we-do/workplace
Health Canada: Mental Health in the Workplace	https://www.canada.ca/en/public-health/services/mental-health-workplace.html
Great-West Life Centre for Mental Health in the Workplace (practical strategies/tools for mental health issues at work)	https://www.workplacestrategiesformentalhealth.com
Guarding Minds @ Work (resources for addressing psychological health & safety in the workplace)	https://www.guardingmindsatwork.ca
Mental Health Works (workplace mental health resources)	http://www.mentalhealthworks.ca
CCOHS Fact Sheet: Return to Work (explanation and components of RTW programs)	https://www.ccohs.ca/oshanswers/psychosocial/return_to_work.html
Canadian Human Rights Commission’s Guide for Managing the Return to Work	http://www.chrc-ccdp.ca/eng/content/guide-managing-return-work
Policy on Duty to Accommodate Persons With Disabilities in the Federal Public Service	http://www.tbs-sct.gc.ca/pol/doc-eng.aspx?id=12541
Institute for Work &Health	https://www.iwh.on.ca/

**Table 20:** Resources for patients on disability and benefit-related programs.

Organization	Link
Provincial Workers’ Compensation Boards in Canada	https://www.ccohs.ca/oshanswers/information/wcb_canada.html
Association of Workers’ Compensation Boards of Canada	http://www.awcbc.org
Benefits Information Kit (for public service employees)	https://www.canada.ca/en/treasury-board-secretariat/topics/benefit-plans.html
Disability Insurance (for public service employees)	https://www.canada.ca/en/treasury-board-secretariat/topics/benefit-plans/plans/disability-insurance-plan.html
Public Service Alliance of Canada (PSAC): Disability Insurance Tips	http://psacbc.com/sites/bc/files/disability-insurance-tips_0.pdf
Employment Insurance Sickness Benefits	http://www.servicecanada.gc.ca/eng/sc/ei/benefits/sickness.shtml

## 7. Future research and conclusion

The assessment and treatment of work-related MDD is a complex process involving patients, clinicians, employers, occupational health, and disability providers. Although major strides have been achieved in this area, challenges remain due in a large part to clinician and patient mistrust of the disability process, limited knowledge awareness, poor communication, and a lack of coordinated and collaborative approaches to return to work or to improve work productivity.

From a clinician perspective, a consensus and GRADE level evidence-based standards of care for the assessment and management of work-related MDD are lacking. Future coordinated research with patients, employers, and researchers, hopefully, will provide much-needed guidance on the appropriate screening tools for the diagnosis of work-related MDD, and functioning instruments to assess more precisely abilities, limitations, and restrictions. Research that provides more clarity and precision on the timelines that are optimal to return to work, duration of the sick note, and the utility of time off work in achieving better outcomes such as functioning or return to work is lacking.

The extant literature regarding antidepressants to treat work-related MDD does not provide any clear choices for clinicians. Although challenging, future medication research around the treatment of work-related MDD must assess workplace function as a primary outcome measure and randomize workers. Research incorporating medications, specific and targeted psychotherapies, workplace interventions, and lifestyle approaches in single or multiple interventions is crucial to personalize the most effective treatment.

Finally, starting in medical school, residency and extending through the years in clinical practice, education should focus on the prevalence and impact of mental health disorders in the workplace. More specifically, this document intends to provide practical recommendations and solutions to a complex clinical problem in the absence of adequate research that is needed to provide confident recommendations. However, the committee hopes that this document is an initial step in establishing a future national standard, with GRADE-like evidence and recommendations to help clinicians manage patients who experience work-related MDD.

## Author contributions

PC drafted the initial manuscript. All authors critically revised the manuscript, gave final approval, and agreed to be accountable for all aspects of the work ensuring integrity and accuracy.

## Funding

This study received funding via arms-length grants to the Workplace Mental Health Network from Allergan Inc., Lundbeck Canada Inc., Janssen Inc., Otsuka Canada Pharmaceutical Inc. and Telus Mobility. The funder was not involved in the study design, collection, analysis, interpretation of data, the writing of this article or the decision to submit it for publication.

## Conflict of interest

PC: Support for the present manuscript from Abbvie, Janssen, Lundbeck, Otsuka, Takeda, and Telus; and consulting fees and payment or honoraria from Abbvie, Elvium, Janssen, Lundbeck, Otsuka, and Takeda. AB: Consulting fees from RBC Insurance, WSIB (Ontario), and the Liquor Control Board of Ontario (LCBO); honoraria for speaking engagement from Osgoode School of Law; payment for expert testimony from the Canadian Medical Malpractice Agency; participation on a data safety monitoring board or advisory board for Lundbeck; and an unpaid leadership or fiduciary role with the Cunningham Society. SB: Consulting fees from Abbvie and Otsuka; payment for lectures or educational events from Janssen-Ortho, Lundbeck, Takeda, Otsuka, and Abbvie; travel support for meeting attendance from Otsuka; and board membership for the Saskatchewan chapter of the Canadian Psychiatric Association. GA: Consulting fees from Abbvie, Allergan, Amgen, Astellas Pharma, AstraZeneca, CCRN, CCPDHM/CHRC, Esai, Galderma, Janssen, Lundbeck, MD Briefcase, MedPlan, Novo Nordisk, Otsuka, Purdue, Takeda, The Academy, Valeant, and Novartis; payment or honoraria for speakers bureaus from Elvium, Sunovion, Esai, Janssen, Takeda, Lundbeck, Valeant, Allergan, Otsuka; and board membership (volunteer) for the Canadian ADHD Resource Alliance (CADDRA). MC: Institutional research grants from the Canadian Institutes of Health Research (CIHR), Social Sciences and Humanities Research Council of Canada (SSHRC), Fonds de recherche du Québec (FRQSC), and funding of a research chair position at the Institut Universitaire en Santé Mentale de Montréal (IUSMM); payment or honoraria from IGF Québec, Les Affaires, Canadian Pension and Benefits Institute (CPBI-ICRA), Swiss Society of Social Psychiatry, University of Montréal, Colloque Insertion Vaud 2022, and AMPQ. DD: Grants or contracts from SSHRC; book royalties from Guilford Press, American Psychological Association, Elsevier, and Pearson; consulting fees from Otsuka; payment or honoraria for presentation from McGill University; board membership for Mental Health Research Canada and the International Association of Applied Psychology; editorial board membership for Clinical Psychology: Science and Practice, Canadian Psychology, Cognitive Therapy and Research, and Canadian Journal of Behavioral Science. JeH: Consulting fees from Otsuka, HLS, Amgen, Pfizer, Eisai, Eli-Lilly, Janssen, Lundbeck, Novo-Nordisk, Valeo, Abbvie, Elvium, and Bausch; and payment or honoraria for speakers bureaus from Pfizer, Amgen, Abbvie, GSK, Bayer, Boehringer, Eli-Lilly, Purdue, Bausch, Allergan, AstraZeneca, Novartis, Lundbeck, Novo-Nordisk, Janssen, Eisai, HLS, Otsuka, Sunovion, MDBriefcase, Liv, MedPlan, Master Clinician Alliance, Academy, Bridge, PeerVoice, Seacourses, Thrombosis Canada, Meducom, Antibody, CHRC, CTC, STA, CCRN, CPD Network, Telus Health, EOCI, AgenceUnik, and Pri-Med. JoH: Employment with Metis Cognition Ltd. Royalties or licenses from Metis Cognition Ltd. for BASIC; consulting fees (paid to Metis Cognition Ltd) from Actinogen, Alzecure, ADvantage, AstraZeneca, Athira Therapeutics, Axoltis, Axon Neuroscience, Bial Biotech, Biogen Idec, Boehringer Ingelheim, Brands2Life, Cerecin, Cognition Therapeutics, Compass Pathways, Corlieve, Curasen, EcoR1, Eisai, EIP Pharma, Eli Lilly, EQT Life, Games for Health EU, Heptares, Ki-Elements, Lundbeck, Lysosome Therapeutics, MyCognition, Neurotrack, NHS, Novartis, Novo Nordisk, Nutricia, Recognify, Remynd, Roche, Rodin Therapeutics, Signant Health, Syndesi Therapeutics, Takeda, Vivoryon Therapeutics, Winterlight Labs, Stoke Therapeutics, Alto Pharma; payment or honoraria (paid to Metis Cognition Ltd) for presentations from Roche; travel support for meeting attendance (paid to Metis Cognition Ltd) from Roche and Biogen; patent for MyCognition; participation on a data safety monitoring board or advisory board for Esai; and stock or stock options from Neurotrack. MK: Grants or contracts from Abbvie, AstraZeneca, Biotics, Canopy, Eisai, Janssen, Lundbeck, Otsuka, Shire, Takeda, and Biohaven; consulting fees from Janssen, Eli Lily, Novartis, Merck, Otsuka, Pfizer, Purdue, Sante Cannabis, Sunovion, Takeda, and Tilray; payment or honoraria from Abbvie, Biron, Alefia Cannabis, Allergan, Bausch Health, Canopy, Eisai, Elvium, Empower Cannabis, Lundbeck; participation on a data safety monitoring board or advisory board for Abbvie, Lundbeck, Biron, Elsai, and Ironshore; board membership for ASPARD; and receipt of equipment, materials, drugs, medical writing, gifts, or other services from Biron. RM: Grants from CIHR/GACD/National Natural Science Foundation of China (NSFC) and the Milken Institute; consulting fees from Lundbeck, Janssen, Alkermes, Neumora Therapeutics, Boehringer Ingelheim, Sage, Biogen, Mitsubishi Tanabe, Purdue, Pfizer, Otsuka, Takeda, Neurocrine, Sunovion, Bausch Health, Axsome, Novo Nordisk, Kris, Sanofi, Eisai, Intra-Cellular, NewBridge Pharmaceuticals, Viatris, Abbvie, and Atai Life Sciences; and is CEO of Braxia Scientific Corp. YL: Institutional grant support from MITACS and Chokka Center for Integrative Health. KN: Institutional grants from the Dutch Social Security Agency, Netherlands Organization for Health Research and Development, and the Dutch Ministry of Education. CD: Consulting fees from Modern Health. Funding Statement: This study received funding via arms-length grants to the Workplace Mental Health Network from Allergan Inc., Lundbeck Canada Inc., Janssen Inc., Otsuka Canada Pharmaceutical Inc. and Telus Mobility. The funder was not involved in the study design, collection, analysis, interpretation of data, the writing of this article or the decision to submit it for publication.

## Publisher’s note

All claims expressed in this article are solely those of the authors and do not necessarily represent those of their affiliated organizations, or those of the publisher, the editors and the reviewers. Any product that may be evaluated in this article, or claim that may be made by its manufacturer, is not guaranteed or endorsed by the publisher.

## Footnotes

^1^ www.pubmed.ncbi.nlm.nih.gov

^2^ https://aced.iwh.on.ca/

^3^Available on The Institute for Work and Health website https://aced.iwh.on.ca/

^4^Available at http://www.mentalhealthroundtable.ca
