# Supplementary material for: Practical pathway for the management of depression in the workplace: a Canadian perspective
Source: Front Psychiatry. 2023 Sep 5;14:1207653. doi: 10.3389/fpsyt.2023.1207653 (PMC10508062; doi:10.3389/fpsyt.2023.1207653)
Supplement: Supplementary file 1 [file Data_Sheet_1.docx]

# Supplement 1: Search terms for pharmacotherapy and psychotherapy for “Practical Pathway for the Management of Depression in the Workplace”

(Final search performed February 2023)

FILTERS: “Human” and “English” Any of: "Clinical Trial, Phase II", "Clinical Trial, Phase III", "Clinical Trial, Phase IV", "Controlled Clinical Trial", "Meta-analysis", "Randomized Controlled Trial", "Systematic Review"

**Work or workplace plus any of the following:**

Antidepressant

Antipsychotic

Bupropion

Citalopram

Desvenlafaxine

Escitalopram

Fluoxetine

Fluvoxamine

Paroxetine

Sertraline

Vilazodone

Vortioxetine

Levomilnacipran

Psychotherapy

Cognitive behavioral therapy

Cognitive behavioural therapy

Cognitive therapy

Mindfulness

Interpersonal psychotherapy

**Cognitive function, cognition plus any of the following:**

Antidepressant

Antipsychotic

Bupropion

Citalopram

Desvenlafaxine

Escitalopram

Fluoxetine

Fluvoxamine

Paroxetine

Sertraline

Vilazodone

Vortioxetine

Levomilnacipran
