# Supplementary material for: Practical pathway for the management of depression in the workplace: a Canadian perspective
Source: Front Psychiatry. 2023 Sep 5;14:1207653. doi: 10.3389/fpsyt.2023.1207653 (PMC10508062; doi:10.3389/fpsyt.2023.1207653)
Supplement: Supplementary file 2 [file Data_Sheet_2.docx]

# Supplement 2: Sample patient cases illustrating clinical situations

## SECTION 3: Assessment and diagnosis of MDD – Work factors contributing to mental health disorders

### Diagnosis of work-related mental health disorders

#### CASE VIGNETTE #1: Amira

Amira, a 27-year-old junior dentist in a large practice presents with a 2-month history of work difficulties. She is experiencing increasing problems with short term memory, concentration and focus, and is making mistakes while charting. She describes low mood/anhedonia, feeling keyed up and on edge, with a tightness in her neck and shoulders, trouble relaxing and shutting her mind off, trouble falling and staying asleep, low energy and motivation, and a lack of interest in her hobbies.

She has missed some days of work, sees fewer patients than usual, and her quality of work has been compromised despite working longer hours to complete charting and to see all scheduled patients. She expresses frustration that her senior colleagues and management have pressured her to see more patients and have been critical of her work performance. At home, she has become more frustrated and avoidant, and claims that work stress has put a strain on her relationship.

On questioning she reports no history of mental health disorders, but says that she thinks her sister is taking “some antidepressant” but she’s not sure. She is otherwise healthy.

*FAQ: How would you assess, diagnose MDD and other mental health disorders, and measure severity and functional impairment in Amira’s case?*

#### CASE VIGNETTE #1: Amira (Continued)

Practical suggestions to screen for and diagnose MDD, and to assess the severity and functional impairment in Amira’s case:

- Amira presents with several months of mood, anxiety, cognitive, and physical symptoms, which are impairing work function. She had similar symptoms while in first year of dental school. Her sister has MDD
- Amira consented to obtaining collateral history from her employer and partner/family
- Her employer indicates a good work history, with no negative evaluations. However, she has had some “disagreements” with a senior dentist who has expressed some concern over Amira’s skill and pace with patients. They deny any “pressure” for her to perform, but have noticed that she has become more withdrawn from her colleagues, has missed work, and is unable to keep up with her patient load. Her employer has reached out to Amira to offer help, but she has told them that “everything is fine”, but she is going through some “personal stress”
- Her husband informs you that the relationship is strong and that he is supportive, but that she has become more reactive, and withdrawn
- On screening, her PHQ-9 is 22 (moderate to severe MDD), GAD-7 is 14 (moderate anxiety), and EarlyDetect has ruled out other comorbid conditions including bipolar disorder, ADHD, alcohol use disorder
- Her SDS total score is 18/30 (moderate functional impairment), with subscale scores for work of 8, social life of 4, and family/home life of 6
- Use clinical judgement and tools to further assess functional and cognitive impairment (Table 1 and 3)
- Based on DSM-5 criteria, clinical evaluation, and screening tools, a diagnosis of MDD is warranted

### Assessing disability or fitness to work

#### CASE VIGNETTE #2: Robert

Robert is a 38-year-old male computer information technologist working at a start-up software company. He has had previous episodes of MDD, but is not currently on any medications, as he has felt well for last year. However, he informs you that over the last 6 months he has begun experiencing a relapse of symptoms that he feels is related to a reduction in the number of hours he is working, financial stress, and possible job layoff associated with the pandemic and the company’s financial struggles. He is highly anxious and is experiencing disrupted sleep, low-energy, sad mood, poor motivation with reduced concentration, easily distracted, poor memory, and problems with multitasking and problem solving. He is making mistakes, procrastinating, and taking longer or unable to complete projects in a timely fashion. Robert’s supervisor has expressed concerns about his mental health and how it’s affecting productivity and customers have complained about receiving poor IT support. Robert feels that his supervisor and employer are not understanding of his mental health symptoms and he feels shame, and stigma in discussing these matters with his employer. He comes to you requesting some time off from work and treatment for his mental health symptoms.

*FAQ: How would you assess Robert’s functioning and fitness to work?*

#### CASE VIGNETTE #2: Robert (Continued)

Practical suggestions to assess Robert’s functioning and fitness to work.

- Obtain a more detailed description of Robert’s job. This will help you to integrate his presenting symptoms and how they impact work function
- If possible, obtain collateral history from Robert’s employer after receiving his consent. If this is not possible question Robert regarding workplace conflicts, work load, productivity, perception of support, any negative evaluations, and previous time off work for similar mental health symptoms and disorder
- Robert’s informs you that he has missed work in the past, and has had been on short term disability on 2 occasions over the last 5 years. He has had some criticism at work, but admits he may be a little over sensitive to feedback.
- Robert has been offered and he has accepted an employee assistance program (EAP). According to the employer, Robert does return from time off and improves at work briefly, but is unable to sustain attendance or work performance
- When you present the employers concerns, Robert does agree that the EAP program helps somewhat but claims that his workload exceeds his capacity and his employer does not provide appropriate support but rather minimizes his concerns
- Robert’s diagnosis should be based on clinical evaluation including work factors, DSM-5, and screening tools
- His functioning should be based on history and screening (Table 1 and 3). The SDS and WHO-DAS indicate moderate functional impairment, and the PDQ-5 indicates moderate to severe cognitive impairment
- Assess Robert’s abilities, limitations, and restrictions
- Although Robert is attending work he clearly has several limitations along the cognitive, emotional, and physical domains of MDD. Some cognitive limitations are associated with symptoms of poor memory, problem solving, leading to making mistakes. His mental health symptoms have impaired his IT job, specifically productivity, he is requiring more time to complete tasks and working longer hours, as well his work performance has received customer complaints and is noticed by his employer. Emotional limitations could include high levels of anxiety and its impact with coworkers and customers. Physical limitations are his fatigue and tiredness which impact his alertness, motivation, and cognitive functions. At this time, he appears to have several limitations which may prevent him from optimal work function.
- Prior to considering a medical note which Robert is requesting, engage with him on the benefits of work, and if possible collaborate in writing with his employer to help him remain at work as long as possible through work accommodations, such as reduced hours, and more work support
- If accommodations are not possible or the limitations are functionally impairing, consider a medical note for work absenteeism
- Keep the medical note time limited (2-4 weeks), focus treatment on functioning, and work collaboratively with Robert, his employer, and his insurer if required
- Focus on Robert returning to work as soon as possible with accommodations or part-time work

## SECTION 4: Pharmacological and psychological treatments for workplace-related mental health disorders

#### CASE VIGNETTE #3: John

John, a 36-year-old father of two boys (8 and 10 y), develops depressive symptoms after a series of difficult life events: including the recent passing of his father and mother. In addition, the company he worked for was recently acquired by another company. New company rules and other changes have made his job more and more difficult. He had become increasingly depressed because of the progressive difficulties on the job. He has low mood and loss of interest, lacks energy, has trouble maintaining sleep, and is especially impaired by intense worry about the future, headaches, chest tightness and episodic panic attacks.

He has been on disability for the last 6 months. He was initially treated with escitalopram (initiated at 5 mg/day and titrated to 20 mg/day). He discontinued the medication after 2 months, complaining of weight gain, fatigue, and cognitive impairment (specifically difficulties in concentration and memory). He also noted difficulties with sex drive and with reaching orgasm. His medication was switched to sertraline (initiated at 25 mg/day and titrated to 100 mg/day). While he noted that he was no longer depressed on the sertraline, he said that he felt flat “with everything feeling blah,” could not concentrate and function, and continued to have an inability to orgasm. He continues to experience worry, feeling keyed up, difficulty in shutting his mind off, tightness in his neck and shoulders, headaches, and bloating.

*FAQ: Are there antidepressant medications that have more evidence for efficacy in the working patient with MDD?*

*FAQ: Are psychotherapy approaches helpful for my patients with work-related mental disorders? What type of therapy works, and what is not beneficial?*

#### CASE VIGNETTE #3: John (Continued)

Applying principles of pharmacotherapeutic and psychotherapeutic treatment to manage John’s work-related MDD.

- There is no consensus regarding the optimal antidepressant for work-related MDD. Select guideline-recommended antidepressants that offer a balance of efficacy and tolerability, and optimize dose and duration
- John needs a treatment that targets all of the dimensions of MDD, bearing in mind that his anxious distress is most impairing
- He was previously treated with 2 different SSRIs, but experienced tolerability issues, and continued to have residual symptoms
- Strategies would include switching medications, or adding an adjunctive agent. John was switched to desvenlafaxine and the dosed optimised to 100 mg/day (vortioxetine, bupropion, or vilazodone are other recommended options)
- John improved, but continued to have residual symptoms of mood, anxiety and fatigue. Brexpiprazole 0.5 mg was initiated and optimised to 2 mg, which led to remission of symptoms and improvement of function
- John would also benefit from CBT, specifically work-based CBT, which has demonstrated improvements in sickness absence and return to work outcomes
- Behavioral activation, nutrition and general stress reducing techniques such as mindfulness have also shown adjunct benefits in treating work-related MDD

## SECTION 5: Integrative treatment strategies for work-related mental health disorders

#### CASE VIGNETTE #4: Julianna

Julianna is a 44-year-old teacher who has been off work for depression for the last 5 months. She has responded to treatment with vortioxetine 15 mg and aripiprazole 4 mg. She completed a 10 week EAP program of supportive, and stress management treatment as well as 15 sessions of work-related CBT. Overall, she rates herself as being 70% of her usual self with improvement in her day-to-day activities, self-care, socialising, and family responsibilities. She has had some pressure from her disability insurer to return to work, and she also informs you that she would like to go back to work. However, she seeks your advice on how to design a successful return to work plan. She is fearful of relapse of symptoms, work demands, and some stigma around her mental health diagnosis.

*FAQ: How do I engage my patient in a goal of recovery and return to work?*

#### CASE VIGNETTE #4: Julianna (Continued)

Strategies to engage Julianna in a goal of recovery and return to work.

- Establish a treatment alliance with Julianna; instill hope and discuss shared goals of recovery and return to work
- Ideally, engage in regular conversations on the importance of work to overall health and wellbeing, and set return-to-work goals early in treatment
- Identify barriers to return to work. Julianna’s symptoms have improved and her functioning is returning. She has some pressure to return to work. She is also motivated to return, but has trepidations
- After obtaining consent from Julianna and being aware of her job description, coordinate an integrated approach with Julianna, her insurer, and her employer
- Provide return date, and discuss work accommodations including number of hours and days to be worked per week initially, duties, and a timeline to return to full-time work
- Identify factors that are treatable with medications and therapy
- Provide regular follow-up, monitoring of symptoms and functioning (SDS), and focus on established goals
- Discuss factors that may be less responsive to treatment and identify referral needs (e.g., job satisfaction, career change, stage of life, intractable work factors, personality traits and disorders, substance use). See Supplemental material S4 (available online).

## SECTION 6: Patient communication/advocacy

#### CASE VIGNETTE #5: Laila

Laila has been a long-term patient in your practice and usually enjoys good health. Unfortunately, she developed post-partum depression and needed to extend her maternity leave much longer than expected. She is now scheduled to return to her demanding position in public relations, which may require working unpredictable hours and engaging with the public in high-profile situations. She loves her job but doesn’t feel ready to take on “everything” given her residual difficulties with emotionality and concerns about relapse and the disruption of her established routine. Laila is also concerned her reputation is at stake if anyone knew that she was not the “strong” woman she has always portrayed. She is unsure how to broach a request for accommodation with her employer.

*FAQ: How do I advocate for my patients in dealing with work place issues, stigma, and discrimination?*

#### CASE VIGNETTE #5: Laila (Continued)

Practical tips to advocate for Laila in dealing with work place issues, stigma, and discrimination

- Advise Laila, who is reluctant to discuss or share any information about mental illness with her workplace, that employers do not need a diagnosis, and she has a right to privacy
- Probe the origin of Laila’s stigma around mental illness. Make her aware that not disclosing her mental health symptoms could pose a barrier to obtaining successful accommodation and support
- Request permission and obtain explicit consent from Laila to obtain clinical records from third-parties such as employers or insurers
- Provide a summary letter instead of records and invoice accordingly, or ask the third-party to send a form for completion (e.g., attending physician statement)
- If Laila is in a dispute with her employer, consider referring her to appropriate services. Stay within your roles and boundaries as a physician. For example, encourage patients to access legal support for legal problems
