# Supplementary material for: Practical pathway for the management of depression in the workplace: a Canadian perspective
Source: Front Psychiatry. 2023 Sep 5;14:1207653. doi: 10.3389/fpsyt.2023.1207653 (PMC10508062; doi:10.3389/fpsyt.2023.1207653)
Supplement: Supplementary file 3 [file Data_Sheet_3.docx]

# SUPPLEMENT 3: CLINICAL TIPS FOR ASSESSING AND MANAGING WORKPLACE-RELATED MDD

**Assessing and diagnosing workplace related MDD and comorbid disorders**

- Diagnose MDD based on DSM-V criteria
- Use patient reported screening tools such as PHQ-9, GAD-7, MDQ, ASRS, and AUDIT to screen for MDD, GAD, bipolar disorder, ADHD, and alcohol use disorder, respectively
- Patients should undergo a routine medical assessment to screen for comorbid medical disorders
- Consider digital screening tools such as EarlyDetect to screen for MDD and comorbid conditions
- Assess cognition using PDQ-5 or other validated tools (see Table 1)
- Consider the assessment of workplace factors (see Table 2), and the timing of work pressures or changes in the workplace relative to symptom onset
- Assess functioning or workplace productivity using SDS and WHO-DAS 2.0 and consider the ODI (the occupational depression inventory) to assess the severity of work-related depressive symptoms (Table 3)
- For assessing disability or fitness to work, please see section 3.4

**Managing workplace related MDD**

- Because of the benefits of work to overall mental and physical health, when possible, encourage patients to stay employed while treating
- Clinicians whenever possible and with patient consent should employ a collaborative model and communicate with various stakeholders to coordinate a return-to-work plan including evaluation of limitations, restrictions, accommodations, and timelines to gradual return to work, at part-time or full-time capacity. In some cases, permanent disability may need to be considered
- Depending on provincial jurisdictions, it is not mandatory to provide a medical note for work absence. However, If the worker requires time off due to MDD and related comorbid conditions, it is suggested initially to provide a medical note that is brief in time (2-4) weeks. This time could provide some clinical evidence whether the patient is able to return to work or be considered for longer work absence. The evaluation of short- and long-term disability is a clinical judgement and encompasses the principles and goals discussed in this paper. In addition to determining the benefits or risks of work absence, it is crucial for the clinician and patient to establish clear treatment goals, regularly monitor symptom and functional outcomes and discuss a timely return to work
- After confirmation of workplace MDD, review and assess treatment options based on patient factors, symptom severity and functional impairments
- For pharmacotherapy there’s no specific medication for work related MDD, but see Supplemental material S4 to guide the selection of the appropriate medication taking into consideration patient factors, tolerability, cognition and functional outcomes
- For psychotherapy Work-CBT, CBT, Problem-solving therapy and combined group and individual therapy show superior effect sizes, please see Table 12 and 13 for more detail

**Additional roles for clinicians in managing workplace related MDD**

###### Helping patients understand their rights

- Workers who report mental health-related impairments to their employers are protected from discrimination under Canadian Human Rights legislation and Canadian Labour Law (see resources below) but in some cases, they may need legal assistance to exercise those rights
- Workers may have access to legal advice and support through their union and other representative associations in achieving appropriate accommodation and maintaining meaningful work duties
- Physicians should be mindful of balancing their roles as medical expert and advocate, with the need to recommend their patients seek outside assistance for workplace disputes
- Physicians should obtain explicit written instruction and consent before communicating with an employer. They should clarify with their patient with whom, and what, should be shared to avoid confidentiality breaches and complaints

###### Helping patients communicate with workplace about their MDD and comorbid disorders

- Obtaining written consent from your patient to communicate with a specified human resources or occupational health professionals on an ongoing basis will enhance the likelihood of cooperation with accommodation recommendations
- Clinicians should be aware of patient’s perceptions of their individual workplace and support their decision on whether to disclose their mental health disorder
- Establishing peer support, formal or informal, should be encouraged as this may greatly decrease anticipatory anxiety with return to work
- Patients can benefit from role-playing or scripting responses to “difficult” questions or situations upon return to work
- Patients should be reminded that disclosing mental health disorders to their employer affords them more rights and protection than not disclosing

###### Helping patients navigate government programs/resources and complete disability forms

- Physicians should inquire with their patients to ensure they have filed a workplace injury claim if a psychological injury occurred at work. If not, they should be reminded to inquire with human resources regarding appropriate forms to complete such as short term disability, long term disability (usually greater than 3 months off work), employment insurance, and sick leave
- Since cognitive issues are common, encourage your patient to keep records and relevant documents and to take notes when any instructions are provided. This will help them stay organized during the process and ensure everyone is responding to the appropriate party on time
- Workers should be encouraged to seek assistance from family, human resources, and their union or employee association to ensure they are completing applications correctly
