# Supplementary material for: Practical pathway for the management of depression in the workplace: a Canadian perspective
Source: Front Psychiatry. 2023 Sep 5;14:1207653. doi: 10.3389/fpsyt.2023.1207653 (PMC10508062; doi:10.3389/fpsyt.2023.1207653)
Supplement: Supplementary file 4 [file Data_Sheet_4.docx]

# Supplement 4: Randomized controlled trials measuring the effects of antidepressants on workplace functioning in MDD

| **Treatment comparison; study** | **Workplace functioning outcome*** |  | **Treatment comparison; study** | **Workplace functioning outcome*** |
| --- | --- | --- | --- | --- |
| **Placebo-controlled RCTs** | | | | |
| **Agomelatine vs. PBO** | |  | **Escitalopram vs. PBO** | |
| Kennedy et al. 2016 (118) | ↓ SDS-Work at week 24 |  | Nierenberg et al. 2007 (119)† | Reduced SDS-Work at week 8, p=NS |
| **Bupropion vs. PBO** | |  | **Levomilnacipran vs. PBO** | |
| Hewett et al. 2009 (120)† | Reduced SDS-Work at week 8, p=NS |  | Asnis et al. 2013 (121) | ↓ SDS-Work at week 8, w/ higher doses |
| Hewett et al. 2010 (122) | ↓ SDS-Work at week 24 |  | Bakish et al. 2014 (123) | ↓ SDS-Work at week 8 |
| **Desvenlafaxine vs. PBO** | |  | Montgomery et al. 2013 (124) | ↓ SDS-Work at week 10 |
| Boyer et al. 2008 (125)† | ↓ SDS-Work at week 8 |  | Sambunaris et al. 2014 (126) | ↓ SDS-Work at week 8 |
| Dunlop et al. 2011 (127) | ↓ SDS-Work at week 12; improved WPAI, p=NS |  | **Paroxetine vs. PBO** | |
| Liebowitz et al. 2008 (128)† | Reduced SDS-Work at week 8, p=NS |  | Detke et al. 2004 (129) | ↓ SDS-Work at week 8 |
| Iwata et al. 2013 (130)† | Reduced SDS-Work at week 8, p=NS |  | Perahia et al. 2006 (131)† | Reduced SDS-Work at week 8, p=NS |
| Liebowitz et al. 2013 (132)† | Reduced SDS-Work at week 8, p=NS |  | **Venlafaxine vs. PBO** | |
| Boyer et al. 2015 (133) | ↓ WPAI at Week 24 |  | Hewett et al. 2009 (120)† | ↓ SDS-Work at week 8 |
| **Duloxetine vs. PBO** | |  | Hewett et al. 2010 (122) | ↓ SDS-Work at week 8 |
| Detke et al. 2004 (129) | Reduced SDS-Work at week 8, p=NS |  | **Vortioxetine vs. PBO** | |
| Perahia et al. 2006 (131)† | Reduced SDS-Work at week 8, p=NS |  | Boulenger et al. 2014 (134) | ↓ SDS-Work at weeks 6 and 8 |
| Nierenberg et al. 2007 (119)† | ↓ SDS-Work at week 8 |  | Mahableshwarkar et al. 2015 (135) | ↓ WLQ-time management |
| Gaynor et al. 2011 (136) | ↓ SDS-Work at week 8 |  | Mahableshwarkar et al. 2015 (137) | Improved WPAI at week 8, p=NS |
| Gaynor et al. 2011 (138) | Reduced SDS-Work at week 8, p=NS |  |  |  |
| Oakes et al. 2012 (139)† | Reduced SDS-Work at week 8, p=NS |  |  |  |
| Boulenger et al. 2014 (134) | ↓ SDS-Work at weeks 6 and 8 |  |  |  |
| Mahableshwarkar et al. 2015 (135) | Reduced WLQ at week 8, p=NS |  |  |  |

#### Supplement 4: continued

| **Active-controlled RCTs** | | | | |
| --- | --- | --- | --- | --- |
| **Agomelatine vs. vortioxetine** | |  | **Fluoxetine vs. sertraline** | |
| Montgomery et al. 2014 (140) | ↓ SDS-Work at weeks 8 and 12 with VOR, p<0.01 |  | Boyer et al. 1998 (141) | ↑ FSQ-Quality of Occupational Life at 6 mos with SER, p=0.025 |
| **Bupropion vs. escitalopram** | |  | **Venlafaxine vs. vortioxetine** | |
| Soczynska et al. 2014 (142) | ↓ SDS-Work and EWPS at week 8. P=NS between agents |  | Wang et al. 2015 (143) | ↓ SDS-Work at week 8. P=NS between agents |
| **Duloxetine vs. escitalopram** | |  |  |  |
| Wade et al. 2007 (144) | ↓ SDS-Work at week 24 with ESC, p<0.05. P=NS between agents at week 8 |  |  |  |

*↓ = significant reduction versus comparator; ↑ = significant increase versus comparator; †Data not reported in original study publication, available in reference (115).

AGO, agomelatine; EWPS, Endicott Work Productivity Scale; FLX, fluoxetine; FSQ, Functional Status Questionnaire; MDD, major depressive disorder; NR, not reported; NS, not significant; PBO, placebo; SDS, Sheehan Disability Scale; SER, sertraline; VOR, vortioxetine; WLQ, Work Limitations Questionnaire; WPAI, Work Productivity and Activity Impairment
