# Supplementary figures and images for: Practical pathway for the management of depression in the workplace: a Canadian perspective
Source: Front Psychiatry. 2023 Sep 5;14:1207653. doi: 10.3389/fpsyt.2023.1207653 (PMC10508062; doi:10.3389/fpsyt.2023.1207653)

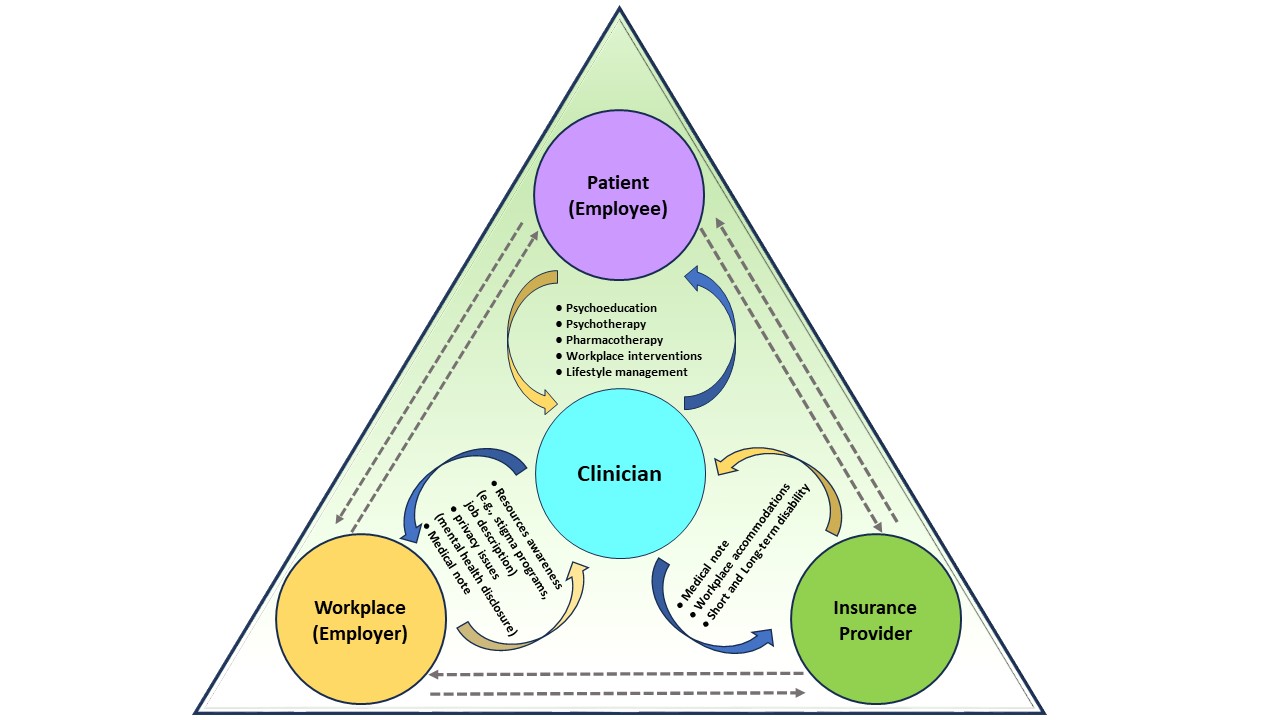

Supplement: Supplementary file 5 [file Image_1.JPEG]
